# Copper-catalyzed domino sequences: a new route to pyrido-fused quinazolinones from 2′-haloacetophenones and 2-aminopyridines†

**DOI:** 10.1039/c8ra03744b

**Published:** 2018-06-04

**Authors:** Phuc H. Pham, Son H. Doan, Ngan T. H. Vuong, Vu H. H. Nguyen, Phuong T. M. Ha, Nam T. S. Phan

**Affiliations:** Faculty of Chemical Engineering, HCMC University of Technology, VNU-HCM 268 Ly Thuong Kiet Street, District 10 Ho Chi Minh City Vietnam ptsnam@hcmut.edu.vn +84 8 38637504 +84 8 38647256 ext. 5681

## Abstract

A new pathway to access pyrido-fused quinazolinones *via* a Cu(OAc)_2_-catalyzed domino sequential transformation between 2′-haloacetophenones and 2-aminopyridines was demonstrated. The solvent and base exhibited a remarkable effect on the transformation, in which the combination of DMSO and NaOAc emerged as the best system. Cu(OAc)_2_·H_2_O was more active towards the reaction than numerous other catalysts. This methodology is new and would be complementary to previous protocols for the synthesis of pyrido-fused quinazolinones.

## Abstract 2

A new pathway to access pyrido-fused quinazolinones *via* a Cu(OAc)_2_-catalyzed domino sequential transformation between 2′-haloacetophenones and 2-aminopyridines was demonstrated.

## Graphical abstract

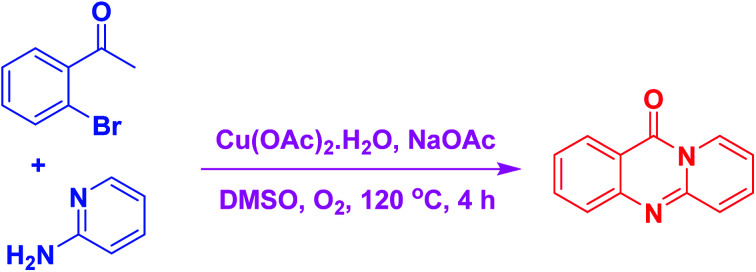

The construction of C–N bonds has attracted significant attention as it is one of the key steps in the synthesis of valuable organic compounds.^1–3^ Quinazolinone derivatives have emerged as a family of privileged structural motifs with a broad spectrum of pharmacological and biological activities.^4–7^ Fused quinazolinones, members of this nitrogen-containing heterocycle family, have particularly gained remarkable interest from the pharmaceutical and medicinal industries.^8,9^ Several efforts have been dedicated to the synthesis of pyrido-fused quinazolinones due to their pharmaceutical value. Chen *et al.* previously synthesized pyrido-fused quinazolinones using a Pd(OAc)_2_-catalyzed carbonylation/intramolecular nucleophilic aromatic substitution transformation.^10^ Sun *et al.* described a CuI-catalyzed tandem reaction *via* aerobic benzylic oxidation, intramolecular cyclization, and decarbonylation of *N*-pyridylarylacetamides to form pyrido-fused quinazolinones.^11^ Rao *et al.* employed Pd(OAc)_2_ and a AgOTf catalyst system for direct aerobic carbonylation with DMF as carbon source to produce pyrido-fused quinazolinones.^12^ Yang *et al.* demonstrated a carbodiimide-mediated condensation of pyridines with anthranilic acids to generate pyrido-fused quinazolinones.^13^ Although interesting results have been achieved, the field still remains to be explored. In this communication, we would like to report a new pathway to pyrido-fused quinazolinones *via* Cu(OAc)_2_-catalyzed domino sequential transformation between 2′-haloacetophenones and 2-aminopyridines.

The reaction between 2′-bromoacetophenone and 2-aminopyridine was explored (Scheme 1). By screening a series of transition metal salts, it was noticed that 11*H*-pyrido[2,1-*b*]quinazolin-11-one was generated as principal product in the presence of a copper salt. Reaction conditions were then screened, utilizing Cu(OAC)_2_·H_2_O as catalyst (Table 1). The reaction was performed in DMSO at 20 mol% catalyst for 4 h, under an oxygen atmosphere, using 2 equivalents of 2-aminopyridine and 2 equivalents of NaOAc as a base. Initially, the influence of temperature on the yield of 11*H*-pyrido[2,1-*b*]quinazolin-11-one was studied (Entries 1–5, Table 1). No product was recorded for the experiment conducted at room temperature. Boosting the temperature led to a remarkable improvement in the yield of the desired product. The most appropriate temperature for the transformation was noted to be 120 °C, with 84% yield being detected (Entry 4, Table 1). The reactant molar ratio displayed a noticeable impact on the reaction, having conducted the reaction with different amounts of 2-aminopyridine (Entries 6–12, Table 1). The reaction utilizing reactant molar ratio of 1 : 1 afforded 64% yield. The yield was upgraded to 90% with 2.5 equivalents of 2-aminopyridine (Entry 9, Table 1). Using excess amounts of 2′-bromoacetophenone resulted in significantly lower yield.

**Scheme 1:**
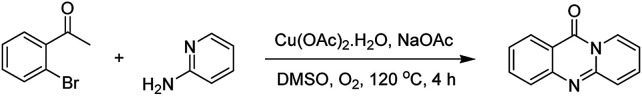
The domino sequential transformation between 2′-bromoacetophenone and 2-aminopyridine.

**Table 1:** Screening of reaction conditions^a^

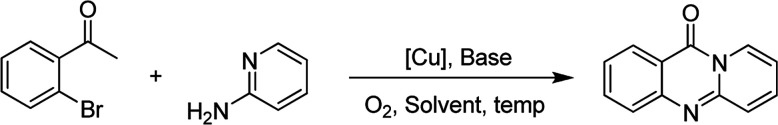						
Entry	Temp (°C)	Reactant ratio (mol : mol)	Catalyst amount (mol%)	Solvent	Base (equiv.)	Yield^b^ (%)
1	RT	1 : 2	20	DMSO	NaOAc (2)	0
2	80					46
3	100					58
4	120					84
5	140					57
6	120	1 : 1	20	DMSO	NaOAc (2)	64
7		1 : 2				84
8		1 : 2.5				90
9		1 : 3				92
10		1.5 : 1				51
11	120	1 : 2.5	0	DMSO	NaOAc (2)	0
12			10			45
13			20			90
14	120	1 : 2.5	20	Toluene	NaOAc (2)	16
15				Dioxane		48
16				DMF		41
17				DMSO		90
18	120	1 : 2.5	20	DMSO	NaOAc (2)	90
19					KOAc (2)	80
20					K_2_CO_3_ (2)	6
21					Piperidine (2)	23
22					Et_3_N (2)	2
23	120	1 : 2.5	20	DMSO	NaOAc (0)	23
24					NaOAc (1)	65
25					NaOAc (2)	90

a Reaction conditions: 2′-bromoacetophenone (0.1 mmol); solvent (0.5 mL); Cu(OAc)_2_·H_2_O catalyst; oxygen atmosphere; 4 h.

b GC yield of 11*H*-pyrido[2,1-*b*]quinazolin-11-one.

One more issue to be investigated for the reaction between 2′-bromoacetophenone and 2-aminopyridine was the catalyst amount (Entries 11–13, Table 1). The reaction was conducted in DMSO at 120 °C for 4 h, under an oxygen atmosphere, using 2.5 equivalents of 2-aminopyridine and 2 equivalents of NaOAc as a base. No trace amount of 11*H*-pyrido[2,1-*b*]quinazolin-11-one was recorded in the absence of Cu(OAc)_2_, verifying the requirement of copper species for the transformation. The best result was achieved for the reaction utilizing 20 mol% catalyst with 90% yield being obtained (Entry 13, Table 1). The reaction was significantly regulated by the solvent, and DMSO emerged as the best solvent for the formation of 11*H*-pyrido[2,1-*b*]quinazolin-11-one (Entry 17, Table 1). A base was required for the reaction, and NaOAc was the base of choice for the system (Entry 18, Table 1). Bulky bases like DBU and *t*BuOK were ineffective for the transformation. The amount of NaOAc also exhibited a remarkable influence on the reaction, and the best yield was observed in the presence of 2 equivalents of NaOAc (Entry 40, Table 1). Nevertheless, expanding the base amount to 3 equivalents resulted in lower yields. Noted that the reaction proceeded to 23% yield in the absence of the base. Moreover, by testing a series of catalyst, Cu(OAc)_2_·H_2_O exhibited better catalytic efficiency over other catalysts in the generation of the quinazolinone (Entry 1, Table 2).

**Table 2:** The reaction between 2′-bromoacetophenone and 2-aminopyridine utilizing different catalysts^a^

Entry	Catalysts	Yield^b^ (%)
1	Cu(OAc)_2_·H_2_O	90
2	Cu(OAc)_2_ anhydrous	79
3	Cu(NO_3_)_2_·2H_2_O	36
4	CuCl_2_·2H_2_O	63
5	Cu(NO_3_)_2_·3H_2_O	74
6	Cu(acac)_2_	29
7	CuBr_2_	64
8	CuBr	75
9	CuI	75
10	Cu powder	58
11	CuSO_4_ anhydrous	36
12	CuO	26
13	Cu_2_O	37
14	Fe(OAc)_2_	0
15	Ni(OAc)_2_·4H_2_O	0
16	Co(OAc)_2_·4H_2_O	0
17	Mn(OAc)_2_·4H_2_O	0

a Reaction conditions: 2′-bromoacetophenone (0.1 mmol); 2-aminopyridine (0.25 mmol); DMSO (0.5 mL); 20 mol% catalyst; oxygen atmosphere; 4 h.

b GC yield of 11*H*-pyrido[2,1-*b*]quinazolin-11-one.

To define the reaction mechanism, several control reactions were performed (Scheme 2). (a) The reaction between 2′-bromoacetophenone (1) and 2-aminopyridine (2) did not occur under an argon atmosphere. (b) The reaction between (1) and (2) did not proceed in the presence of 20 mol% ascorbic acid as antioxidant (c) The reaction between (1) and (2) in the absence of base under standard reaction conditions provided the desired product (3) in 6% yield. Additionally, 27% yield of 2-bromo-*N*-(pyridin-2-yl)benzamide (4) was observed under these conditions (d) The reaction between 2-bromobenzaldehyde (5) and (2) under standard condition did not afford (3), while 36% yield of (4) was observed. (e) The reaction between 2-bromobenzoic acid (6) and (2) under standard condition offered 45% yield of (3), suggesting that (6) could be the key intermediate during the formation of quinazolinone. (f) 72% yield of (3) was obtained for the reaction between 2-(2-bromophenyl)-2-oxoacetaldehyde (7) and (2) under standard condition, proposing that (7) was also formed in the catalytic cycle. (g) The reaction between isatin (8) and 2-bromopyridine (9) under standard condition generated (3) in 75% yield. (h) Heating 1-(2-(pyridin-2-ylamino)phenyl)ethan-1-one (10), Ullmann–Goldberg coupling product between (1) and (2), under standard condition also afforded (3) in 81% yield.

**Scheme 2:**
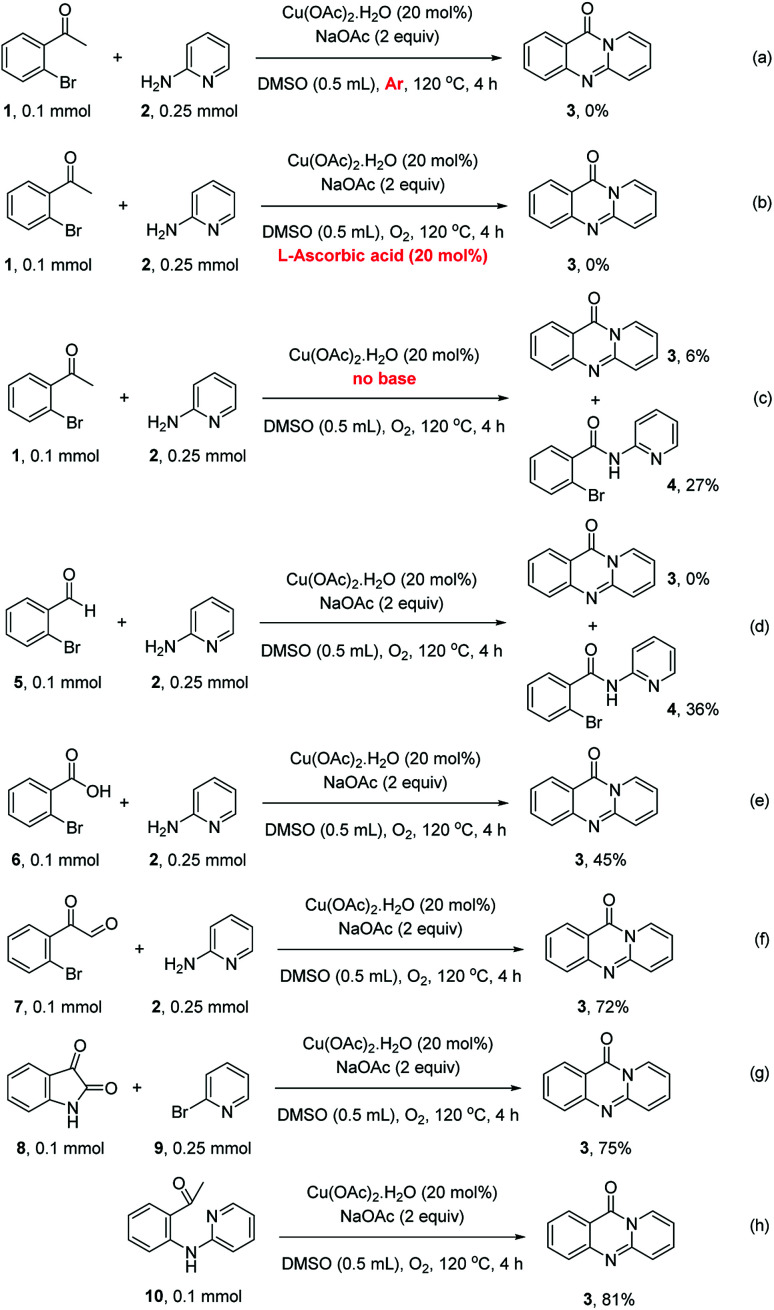
Control experiments.

On the basis of the above results and previously reported works.^14–19^ the reaction pathway was proposed (Scheme 3). Initially, (1) was transformed to (7) *via* the formation of peroxy-Cu(ii) radical B, peroxy-Cu(i) complex C, and dioxetane intermediate D. Subsequently, an Ullmann–Goldberg coupling between (7) and (2) occurred to produce 2-oxo-2-(2-(pyridin-2-ylamino)phenyl)acetaldehyde E. Additionally, E could be generated from (10) *via* similar copper-catalyzed oxidation sequences.^19,20^ Next, E was converted to F in the presence of copper catalyst, and under oxygen (Path I). Indeed, the formation of F from (10) was previously reported by Ilangovan and Satish.^14,15^ Upon base-mediated hydrolysis, G was generated, and the consequent intramolecular addition cyclization occurred to form H. In the next step, decarboxylation and oxidation occurred to form the desired product (3) in the presence of copper catalyst and oxygen. Certainly, the conversion of F to the quinazolinone was demonstrated by Liu *et al.*^16^ For Path II, the hydration of (7) and subsequent 1,2-hydride shift led to the formation of anionic intermediate J. Consequently, decarboxylation occurred to produce (5), and (5) was oxidized to (6) in the presence of copper catalyst and oxygen.^17,21^ Intermediate K was formed *via* an Ullmann–Goldberg coupling, and the consequent amidation cyclization occurred to furnish the desired quinazolinone (3). It should be noted that this amidation cyclization was previously mentioned by Pellón *et al.*^18^ Noted that 45% yield of (3) was obtained for the reaction between (6) and (2). Therefore, the desired quinazolinone (3) would also be produced Path II, though Path I would be more favored.

**Scheme 3:**
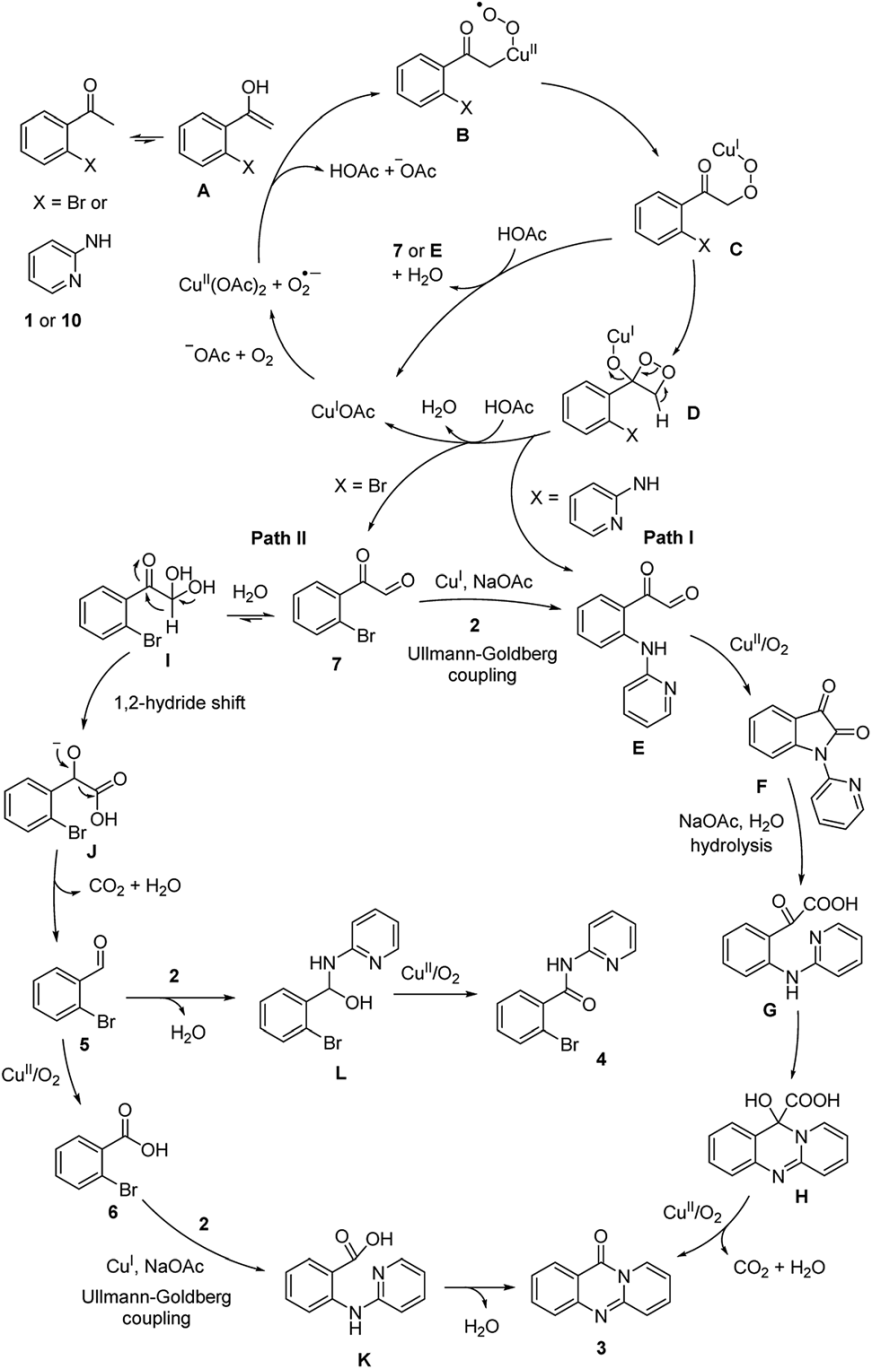
Proposed reaction pathway.

The scope of this work was subsequently extended to the synthesis of several pyrido-fused quinazolinones from different 2′-haloacetophenones and 2-aminopyridines (Table 3). The reaction was conducted in DMSO at 120 °C for 4 h, under an oxygen atmosphere, in the presence of 20 mol% Cu(OAC)_2_ catalyst, using 2.5 equivalents of 2-aminopyridine and 2 equivalents of NaOAc as a base. Quinazolinones were consequently isolated column chromatography. Utilizing this approach, pyrido-fused quinazolinones were produced in high yields. 11*H*-pyrido[2,1-*b*]quinazolin-11-one was achieved in 87% yield *via* the reaction between 2′-bromoacetophenone and 2-aminopyridine (Entry 1, Table 3). Lower yields were recorded for 2-aminopyridines containing a substituent. 6-Methyl-11*H*-pyrido[2,1-*b*]quinazolin-11-one (Entry 2, Table 3) was generated in 68%, while 7-methyl-11*H*-pyrido[2,1-*b*]quinazolin-11-one (Entry 3, Table 3) was formed in 72%. Similarly, 8-methyl-11*H*-pyrido[2,1-*b*]quinazolin-11-one (Entry 4, Table 3) and 9-methyl-11*H*-pyrido[2,1-*b*]quinazolin-11-one (Entry 5, Table 3) were produced in 82% and 71% yields, respectively. Moving to 2-aminopyridines containing a halo substituent, 8-chloro-11*H*-pyrido[2,1-*b*]quinazolin-11-one (Entry 6, Table 3) and 8-bromo-6-methyl-11*H*-pyrido[2,1-*b*]quinazolin-11-one (Entry 7, Table 3) were achieved in 74% and 55% yields, respectively. Furthermore, 2′-iodoacetophenone demonstrated greater reactivity over the 2′-bromoacetophenone, affording the corresponding products in higher yields (Entry 9, 10, 11, 12, Table 3). Noticeably, this method can be applicable to the concise construction of 12*H*-benzo[4,5]thiazolo[2,3-*b*]quinazolin-12-ones. By utilizing 2′-bromoacetophenone and 2′-iodoacetophenone in the reaction with benzo[*d*]thiazol-2-amine under standard reaction conditions, the corresponding products were achieved in 57% and 63% yields, respectively (Entry 8, 11, Table 3). It should be noted that 2-aminopyridines containing strong electron-withdrawing groups such as SO_3_H, CN, and NO_2_ were unreactive for this transformation (Table S8†).

**Table 3:** The synthesis of pyrido-fused quinazolinones from 2′-haloacetophenones and 2-aminopyridines^a^

Compound	Yield^f^ (%)	Compound	Yield^f^ (%)
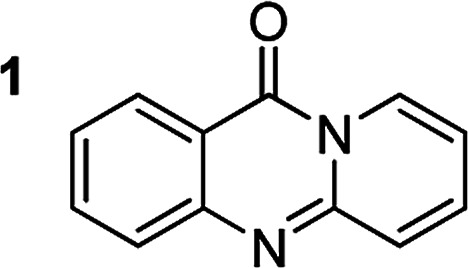	87	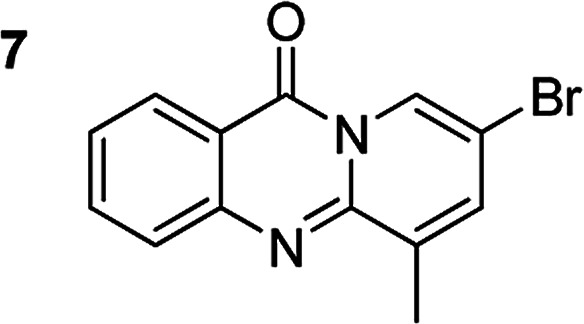	55
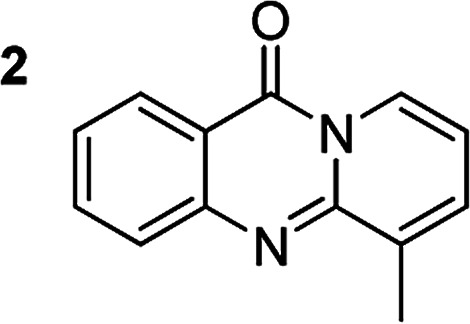	68	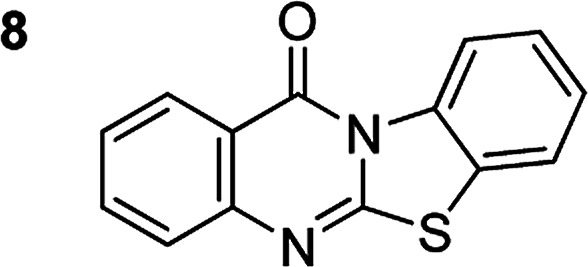	57
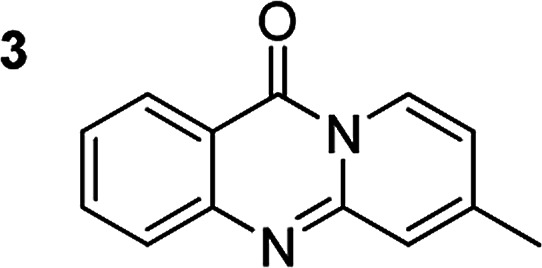	72	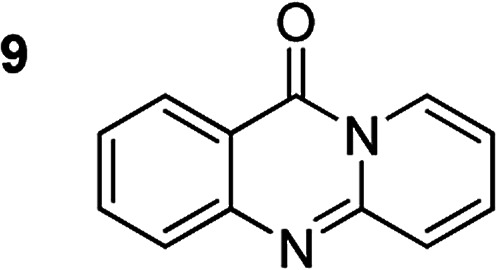	89^b^
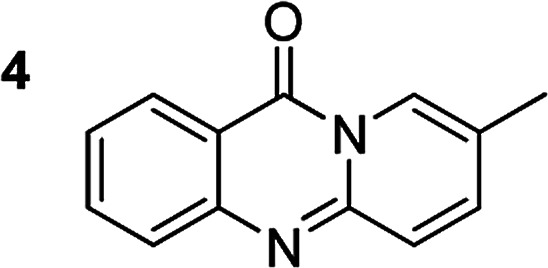	82	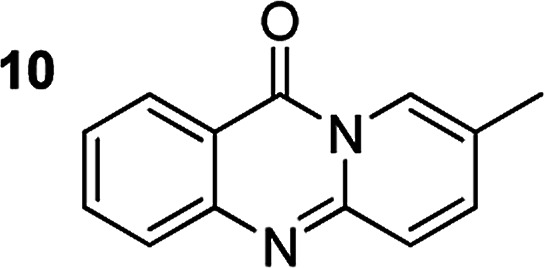	87^c^
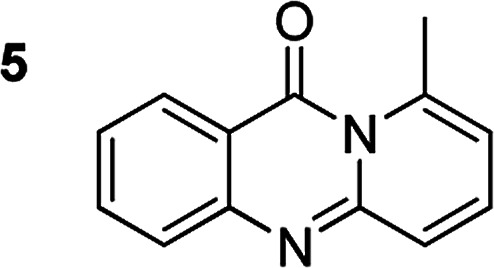	71	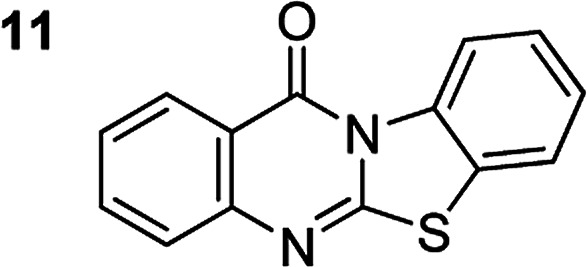	63^d^
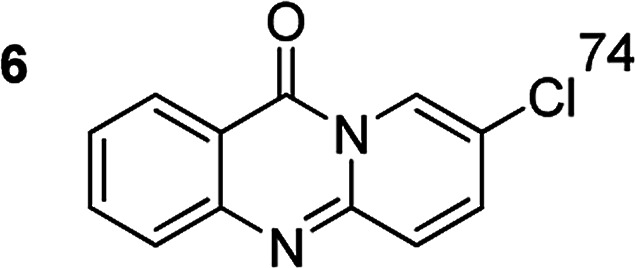	74	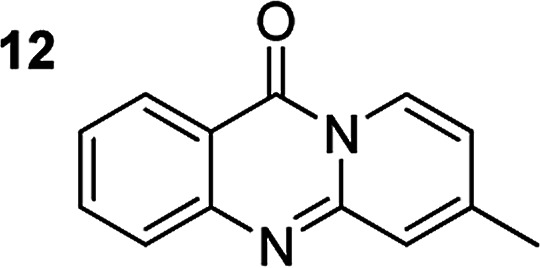	78^e^

a Reaction conditions: 2′-bromoacetophenone (0.1 mmol); 2-aminopyridines (0.25 mmol); NaOAc (0.2 mmol); DMSO (0.5 mL); Cu(OAc)_2_·H_2_O catalyst (20 mol%); oxygen atmosphere; 120 °C; 4 h.

b 2′-Iodoacetophenone (0.1 mmol); 2-aminopyridine (0.25 mmol).

c 2′-Iodoacetophenone (0.1 mmol); 2-amino-5-methylpyridine (0.25 mmol).

d 2′-Iodoacetophenone (0.1 mmol); 2-aminobenzothiazole (0.25 mmol).

e 2′-Iodoacetophenone (0.1 mmol); 2-amino-4-methylpyridine (0.25 mmol).

f Isolated yield.

In conclusion, a new pathway to achieve pyrido-fused quinazolinones *via* Cu(OAC)_2_-catalyzed domino sequential transformation between 2′-haloacetophenones and 2-aminopyridines were demonstrated. The transformation proceeded under an oxygen atmosphere, in the presence of a base. The reaction was remarkably regulated by the solvent and the base, in which the combination of DMSO and NaOAc emerged as the best system for the generation of pyrido-fused quinazolinones. Cu(OAC)_2_·H_2_O was more active towards the reaction than a series of catalysts. Two plausible reaction pathways were suggested. The noticeable advantages of this method are the (1) available starting materials; (2) excellent yields of desired product with low cost catalyst Cu(OAc)_2_·H_2_O; and (3) broad substrate scope. This methodology would be complementary to previous synthetic protocols, and would be interested to the pharmaceutical and chemical industries. Further investigations on the reaction mechanism and on substrate scope are currently underway in our laboratory.

## Conflicts of interest

There are no conflicts to declare.

## Supplementary Material

RA-008-C8RA03744B-s001

## References

[cit1] Wei W. T., Zhu W. M., Ying W. W., Wang Y. N., Bao W. H., Gao L. H., Luo Y. J., Liang H. (2017). Adv. Synth. Catal..

[cit2] Wei W.-T., Zhu W.-M., Bao W.-H., Chen W.-T., Huang Y.-L., Gao L.-H., Xu X.-D., Wang Y.-N., Chen G.-P. (2018). ACS Sustainable Chem. Eng..

[cit3] Wei W.-T., Zhu W.-M., Liang W., Wu Y., Huang H.-Y., Huang Y.-L., Luo J., Liang H. (2017). Synlett.

[cit4] Gholap A. V. A., Maity S., Schulzke C., Maiti D., Kapdi A. R. (2017). Org. Biomol. Chem..

[cit5] Shagufta, Ahmad I. (2017). Med. Chem. Commun..

[cit6] Hou J., Kovacs M. S., Dhanvantari S., Luyt L. G. (2018). J. Med. Chem..

[cit7] Singh J. B., Mishra K., Gupta T., Singh R. M. (2018). New J. Chem..

[cit8] Feng Y., Tian N., Li Y., Jia C., Li X., Wang L., Cui X. (2017). Org. Lett..

[cit9] Mishra A., Mukherjee U., Vats T. K., Deb I. (2018). J. Org. Chem..

[cit10] Chen J., Natte K., Spannenberg A., Neumann H., Langer P., Beller M., Wu X.-F. (2014). Angew. Chem., Int. Ed..

[cit11] Sun J., Tan Q., Yang W., Liu B., Xu B. (2014). Adv. Synth. Catal..

[cit12] Rao D. N., Rasheed S., Das P. (2016). Org. Lett..

[cit13] Yang Y., Zhu C., Zhang M., Huang S., Lin J., Pan X., Su W. (2016). Chem. Commun..

[cit14] Ilangovan A., Satish G. (2013). Org. Lett..

[cit15] Ilangovan A., Satish G. (2014). J. Org. Chem..

[cit16] Liu M., Shu M., Yao C., Yin G., Wang D., Huang J. (2016). Org. Lett..

[cit17] Liu H., Wang M., Li H., Luo N., Xu S., Wang F. (2017). J. Catal..

[cit18] Pellón R. F., Martín A., Docampo M. L., Mesa M. (2006). Synth. Commun..

[cit19] Huang J., Mao T., Zhu Q. (2014). Eur. J. Org. Chem..

[cit20] Borah S., Melvin M. S., Lindquist N., Manderville R. A. (1998). J. Am. Chem. Soc..

[cit21] Wang M., Lu J., Li L., Li H., Liu H., Wang F. (2017). J. Catal..

