# Supplementary material for: Copper-catalyzed domino sequences: a new route to pyrido-fused quinazolinones from 2′-haloacetophenones and 2-aminopyridines
Source: RSC Adv. 2018 Jun 4;8(36):20314–8. doi: 10.1039/c8ra03744b (PMC9080829; doi:10.1039/c8ra03744b)
Supplement: RA-008-C8RA03744B-s001 [file RA-008-C8RA03744B-s001.pdf]

Electronic Supplementary Material (ESI) for RSC Advances.
This journal is © The Royal Society of Chemistry 2018

# Copper-catalyzed domino sequences: A new route to pyrido-fused quinazolinones from 2'-haloacetophenones and 2-aminopyridines

Phuc H. Pham, Son H. Doan, Ngan T. H. Vuong, Vu H. H. Nguyen, Phuong T. M. Ha, Nam T. S. Phan*

Faculty of Chemical Engineering, HCMC University of Technology, VNU-HCM, 268 Ly Thuong Kiet, District 10, Ho Chi Minh City, Viet Nam

*Email: ptsnam@hcmut.edu.vn

Ph: (+84 8) 38647256 ext. 5681 Fx: (+84 8) 38637504

## Supporting information

*Materials and instrumentation*

All reagents and starting materials were obtained commercially from Sigma-Aldrich and Merck, and were used as received without any further purification unless otherwise noted. Gas chromatographic (GC) analyses were performed using a Shimadzu GC 2010-Plus equipped with a flame ionization detector (FID) and an SPB-5 column (length = 30 m, inner diameter = 0.25 mm, and film thickness = 0.25 μm). The temperature program for GC analysis held samples at 120 $^{o}C$ for 0.5 min; heated them from 120 to 130 $^{o}C$ at 40 $^{o}C/min$; held them at 130 $^{o}C$ for 1 min; heated them from 130 to 280 $^{o}C$ at 40 $^{o}C/min$; and finally held them at 280 $^{o}C$ for 1.5 min. Inlet and detector temperatures were set constant at 280 $^{o}C$. The GC yield was calculated using diphenyl ether as the internal standard. GC-MS analyses were analyzed on a Shimadzu GCMS-QP2010Ultra with a ZB-5MS column

(length = 30 m, inner diameter = 0.25 mm, and film thickness = 0.25 μm). The temperature program for GC-MS analysis held samples at 50 °C for 2 min; heated samples from 50 to 280°C at 10 °C/min and held them at 280 °C for 10 min. Inlet temperature was set constant at 280 °C. MS spectra were compared with the spectra gathered in the NIST library. The $^{1}H$ NMR and $^{13}C$ NMR were recorded on Bruker AV 500 spectrometers using residual solvent peak as a reference.

*Experimental procedure*

To a 12-mL screw-cap vial containing DMSO (0.5 mL) was added 2'-bromoacetophenone (19.9 mg, 0.1 mmol), 2-aminopyridines (23.5 mg, 0.25 mmol), anhydrous NaOAc (16.4 mg, 0.2 mmol), $Cu(OAc)_2.H_2O$ (4.0 mg, 0.02 mmol) and diphenyl ether (0.1 mmol) as an internal standard. The catalyst concentration was calculated with respect to the copper/2'-bromoacetophenone molar ratio. The reactor was evacuated and back-filled with oxygen. The resulting mixture was then stirred at 120 °C for 4 h. After that, the mixture was slowly cooled to room temperature, then distilled water (5 mL) was added. The organic components were extracted with dichloromethane (20 mL), dried over anhydrous $Na_2SO_4$ and concentrated under vacuum. The crude product was purified by column chromatography on silica gel with hexane/ethyl acetate as eluent to give pure product. The product identity was further confirmed by GC-MS, $^{1}H$ NMR and $^{13}C$ NMR. The reaction yield was monitored by withdrawing aliquots from the reaction mixture, quenched with brine and the organic components were then extracted into ethyl acetate (2 mL), dried over anhydrous $Na_2SO_4$ and analyzed by GC with reference to diphenyl ether.

**Table S1: Effect of varied temperature on the yield of 11H-pyrido[2,1-b]quinazolin-11-one:**

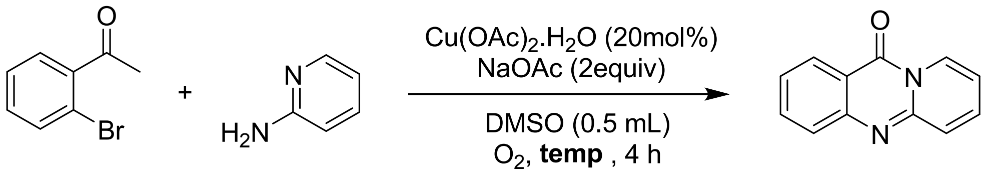


| Entry | Temperature (°C) | Yield[b] (%) |
|---|---|---|
| 1 | RT | 0 |
| 2 | 80 | 46 |
| 3 | 100 | 58 |
| **4** | **120** | **84** |
| 5 | 140 | 57 |

[a]Reaction conditions: 2-bromoacetophenone (0.1 mmol); 2-aminopyridine (0.2mmol); DMSO (0.5 mL); $Cu(OAc)_2.H_2O$ (20 mol%); NaOAc (2 equiv); **temp**; oxygen atmosphere; 4 h.[b] GC yield of 11H-pyrido[2,1-b]quinazolin-11-one.

**Table S2: Effect of diverse reactant mole proportions on the yield of 11H-pyrido[2,1-b]quinazolin-11-one:**

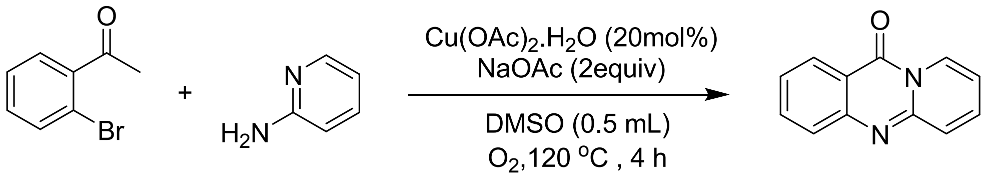


| Entry | Temperature (°C) | Yield[b] (%) |
|---|---|---|
| 1 | 1:1 | 64 |
| 2 | 1:1.5 | 75 |
| 3 | 1:2 | 84 |
| **4** | **1:2.5** | **90** |
| 5 | 1:3 | 92 |
| 6 | 1.5:1 | 51 |
| 7 | 2:1 | 33 |

[a]Reaction conditions: 2-bromoacetophenone (0.1 mmol); DMSO (0.5 mL); $Cu(OAc)_2.H_2O$ (20 mol%); NaOAc (2 equiv); 120 °C; oxygen atmosphere; 4 h.[b] GC yield of 11H-pyrido[2,1-b]quinazolin-11-one.

**Table S3: Effect of different catalyst amounts on the yield of 11H-pyrido[2,1-b]quinazolin-11-one:**

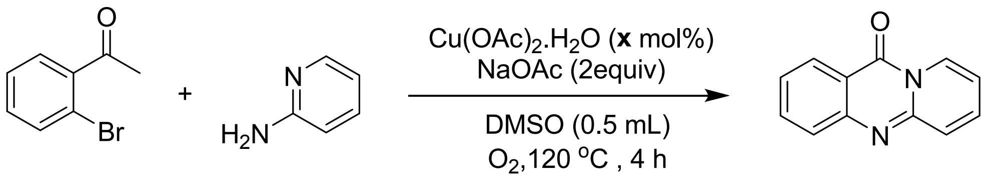


| Entry | Catalyst amount (mol%) | Yield[b] (%) |
|---|---|---|
| 1 | 0 | 0 |
| 2 | 5 | 39 |
| 3 | 10 | 45 |
| 4 | 15 | 67 |
| **5** | **20** | **90** |

[a]Reaction conditions: 2-bromoacetophenone (0.1 mmol); 2-aminopyridine (0.25 mmol); DMSO (0.5 mL); $Cu(OAc)_2.H_2O$ **(x mol%)**; NaOAc (2 equiv); 120 °C; oxygen atmosphere; 4 h.[b] GC yield of 11H-pyrido[2,1-b]quinazolin-11-one.

**Table S4: Effect of diverse solvents on the yield of 11H-pyrido[2,1-b]quinazolin-11-one:**

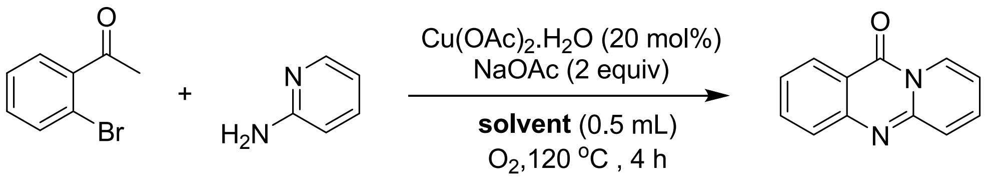


| Entry | Solvent | Yield[b] (%) |
|---|---|---|
| 1 | Toluene | 16 |
| 2 | Xylene | 46 |
| 3 | Dioxane | 48 |
| 4 | Diglyme | 63 |
| 5 | NMP | 75 |
| 6 | DMF | 41 |
| 7 | DMAc | 61 |
| **8** | **DMSO** | **90** |
| 9 | DEG | 25 |

[a]Reaction conditions: 2-bromoacetophenone (0.1 mmol); 2-aminopyridine (0.25 mmol); **solvent** (0.5 mL); $Cu(OAc)_2.H_2O$ (20 mol%); NaOAc (2 equiv); 120 °C; oxygen atmosphere; 4 h.[b] GC yield of 11H-pyrido[2,1-b]quinazolin-11-one.

**Table S5: Effect of different bases on the yield of 11H-pyrido[2,1-b]quinazolin-11-one:**

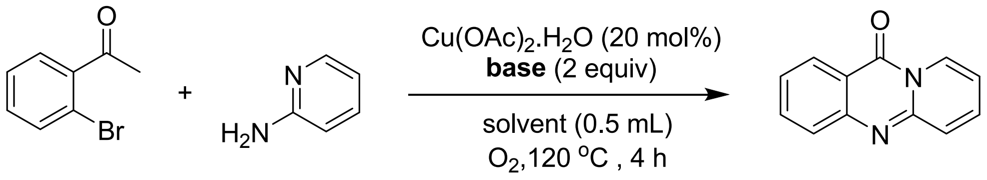

| Entry | Base | Yield[b] (%) |
|---|---|---|
| **1** | **NaOAc** | **90** |
| 2 | KOAc | 80 |
| 3 | $NH_4OAc$ | 14 |
| 4 | $NaHCO_3$ | 19 |
| 5 | $K_2CO_3$ | 6 |
| 6 | $K_3PO_4$ | 4 |
| 7 | KOH | 8 |
| 8 | Piperidine | 23 |
| 9 | $Et_3N$ | 2 |
| 10 | DBU | 0 |
| 11 | Pyridine | 11 |
| 12 | DABCO | 7 |
| 13 | tBuOK | 3 |
| 14 | CsF | 0 |
| 15 | $Cs_2CO_3$ | 0 |

[a]Reaction conditions: 2-bromoacetophenone (0.1 mmol); 2-aminopyridine (0.25 mmol); DMSO (0.5 mL); $Cu(OAc)_2.H_2O$ (20 mol%); **base** (2 equiv); 120 $^oC$; oxygen atmosphere; 4 h.[b] GC yield of 11H-pyrido[2,1-b]quinazolin-11-one.

**Table S6: Effect of varied base amounts on the yield of 11H-pyrido[2,1-b]quinazolin-11-one:**

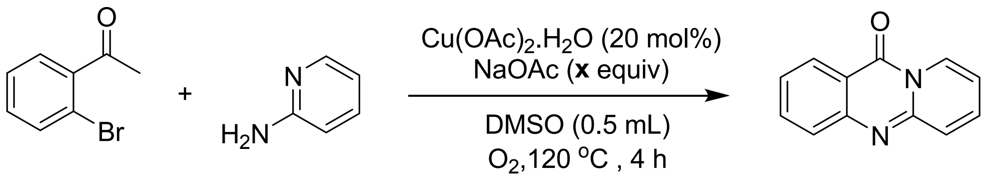


| Entry | Base amount (equiv.) | Yield[b] (%) |
|---|---|---|
| 1 | 0 | 23 |
| 2 | 0.5 | 43 |
| 3 | 1 | 65 |
| 4 | 1.5 | 81 |
| **5** | **2** | **90** |
| 6 | 2.5 | **77** |
| 7 | 3 | 76 |

[a]Reaction conditions: 2-bromoacetophenone (0.1 mmol); 2-aminopyridine (0.25 mmol); DMSO (0.5 mL); $Cu(OAc)_2.H_2O$ (20 mol%); NaOAc (**x** equiv); 120 °C; oxygen atmosphere; 4 h.[b] GC yield of 11H-pyrido[2,1-b]quinazolin-11-one.

**Table S7: Effect of different catalysts on the yield of 11H-pyrido[2,1-b]quinazolin-11-one:**

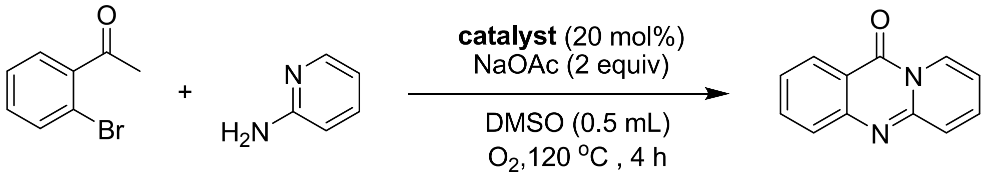


| Entry | Catalysts | Yield[b] (%) |
|---|---|---|
| **1** | **$Cu(OAc)_2.H_2O$** | **90** |
| 2 | $Cu(OAc)_2$ anhydrous | 79 |
| 3 | $Cu(NO_3)_2.2H_2O$ | 36 |
| 4 | $CuCl_2.2H_2O$ | 63 |
| 5 | $Cu(NO_3)_2.3H_2O$ | 74 |
| 6 | $Cu(acac)_2$ | 29 |
| 7 | $CuBr_2$ | 64 |
| 8 | CuBr | 75 |
| 9 | CuI | 75 |
| 10 | Cu powder | 58 |
| 11 | $CuSO_4$ anhydrous | 36 |
| 12 | CuO | 26 |
| 13 | $Cu_2O$ | 37 |
| 14 | $Fe(OAc)_2$ | 0 |
| 15 | $Ni(OAc)_2.4H_2O$ | 0 |

| | | |
|---|---|---|
| 16 | $Co(OAc)_2.4H_2O$ | 0 |
| 17 | $Mn(OAc)_2.4H_2O$ | 0 |

[a]Reaction conditions: 2-bromoacetophenone (0.1 mmol); 2-aminopyridine (0.25 mmol); DMSO (0.5 mL); **catalyst** (20 mol%); NaOAc (2 equiv); 120 $^{o}C$; oxygen atmosphere; 4 h.[b] GC yield of 11H-pyrido[2,1-b]quinazolin-11-one.

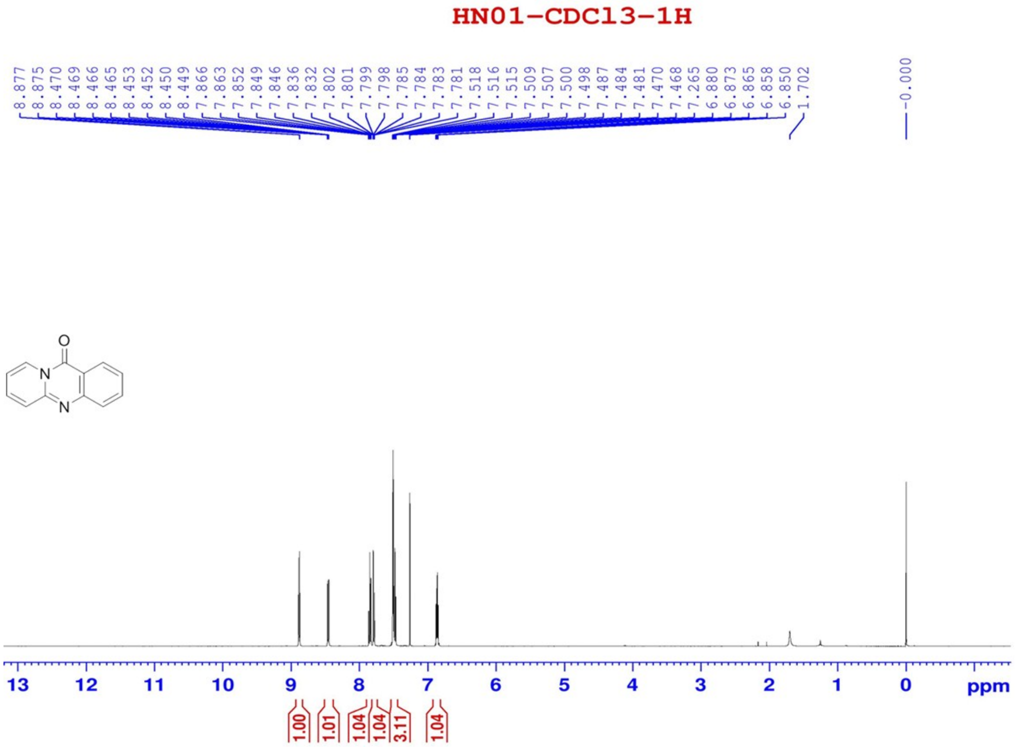


Fig.S1. $^{1}$H-NMR spectra of 11*H*-pyrido[2,1-*b*]quinazolin-11-one.

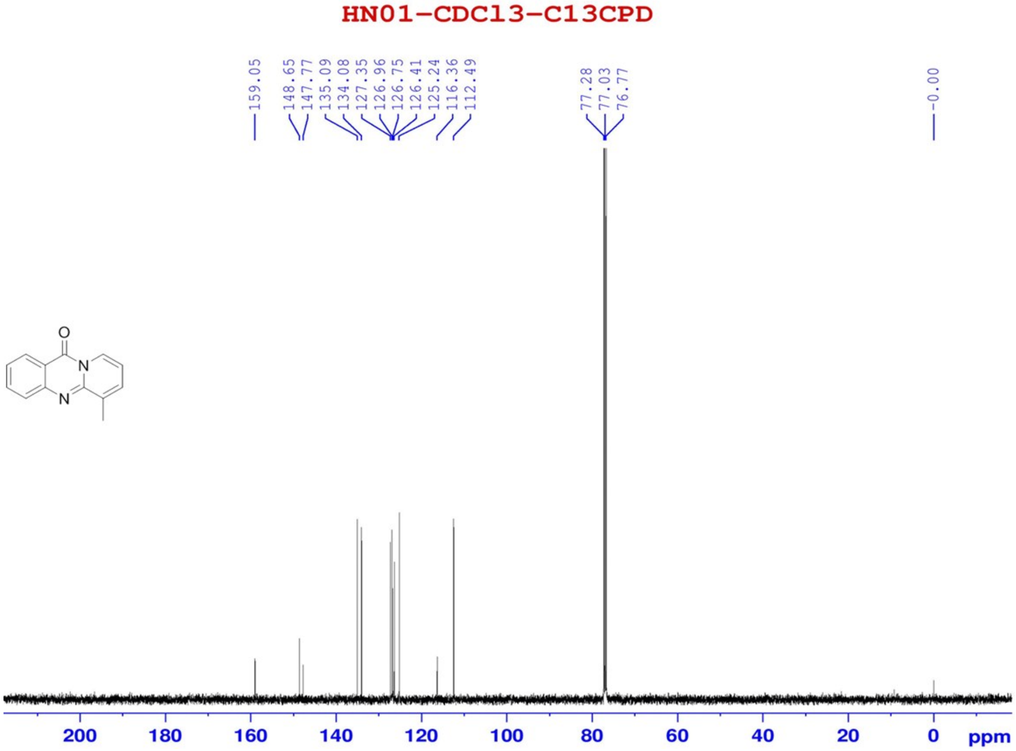


Fig.S2. $^{13}$C-NMR spectra of 11*H*-pyrido[2,1-*b*]quinazolin-11-one.

**Characterization data for 11*H*-pyrido[2,1-*b*]quinazolin-11-one**

Prepared as shown in the general experimental procedure and purified on silica gel (230-400 mesh or 37-63 μm, ethyl acetate/hexane = 1:1 (v./v.), TLC silica gel 60 $F_{254}$, $R_f$ = 0.4): Yellow solid, 87% yield (17 mg).$^1$H-NMR (500 MHz, $CDCl_3$) δ(ppm) 6.85–6.88 (m, 1H), 7.46–7.51 (m, 3H), 7.78-7.86 (m, 2H), 8.44-8.47 (m, 1H), 8.88 (dt, *J* = 7.5 Hz, 1.0 Hz, 1H). $^{13}$C NMR ($CDCl_3$, 125 MHz) δ(ppm) 112.4, 116.3, 125.2, 126.4, 126.7, 126.9, 127.3, 134.0, 135.0, 147.7, 148.6, 159.0. HRMS calcd for $C_{12}H_8N_2O$ $(M+H)^+$: 197.0709, found $(M+H)^+$: 197.0701.

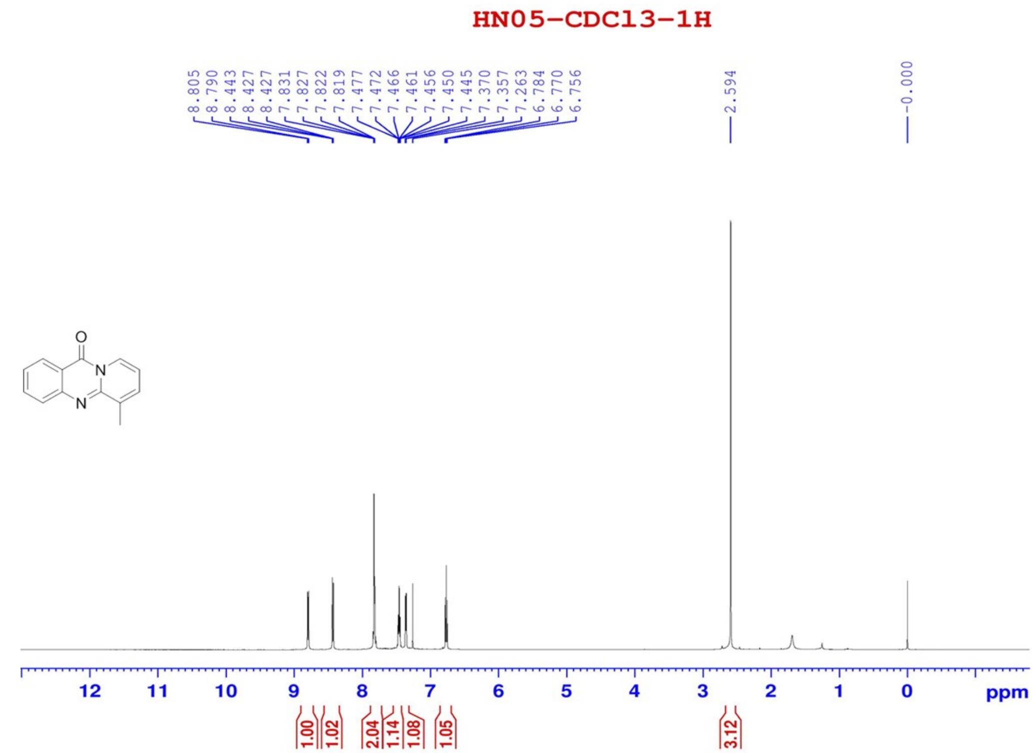


Fig.S3. $^{1}$H-NMR spectra of 6-methyl-11*H*-pyrido[2,1-*b*]quinazolin-11-one.

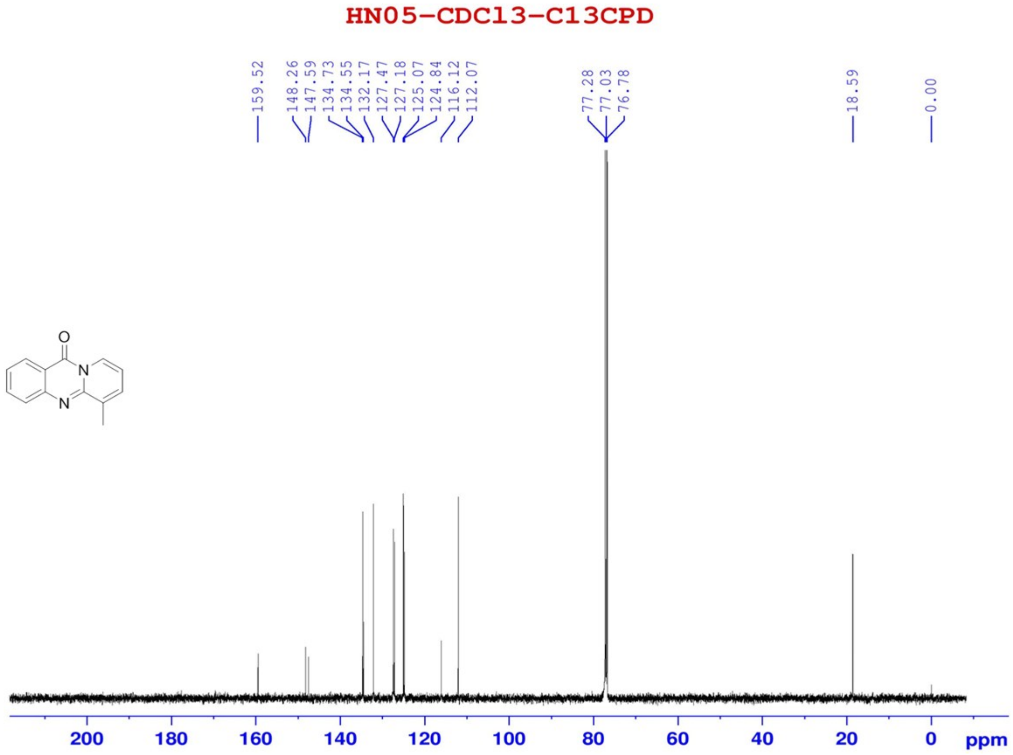

Fig.S4. $^{13}$C-NMR spectra of 6-methyl-11*H*-pyrido[2,1-*b*]quinazolin-11-one.

**Characterization data for 6-methyl-11*H*-pyrido[2,1-*b*]quinazolin-11-one**

Prepared as shown in the general experimental procedure and purified on silica gel (230-400 mesh or 37-63 μm, ethyl acetate/hexane = 1:1 (v./v.), TLC silica gel 60 $F_{254}$, $R_f$ = 0.45): Yellow solid, 68% yield (14 mg). $^1$H-NMR (500 MHz, $CDCl_3$) δ(ppm) 2.59 (s, 3H), 6.77 (t, J = 7.0 Hz, 1H), 7.36 (d, J = 6.5 Hz, 1H), 7.44–7.47 (m, 1H), 7.81–7.83 (m, 2H), 8.43 (d, J = 8.0 Hz, 1H), 8.80 (d, J = 7.5 Hz, 1H).$^{13}$C NMR ($CDCl_3$, 125 MHz) δ(ppm) 18.5, 112.0, 116.1, 124.8, 125.0, 127.1, 127.4, 132.1, 134.5, 134.7, 147.5, 148.2, 159.5.

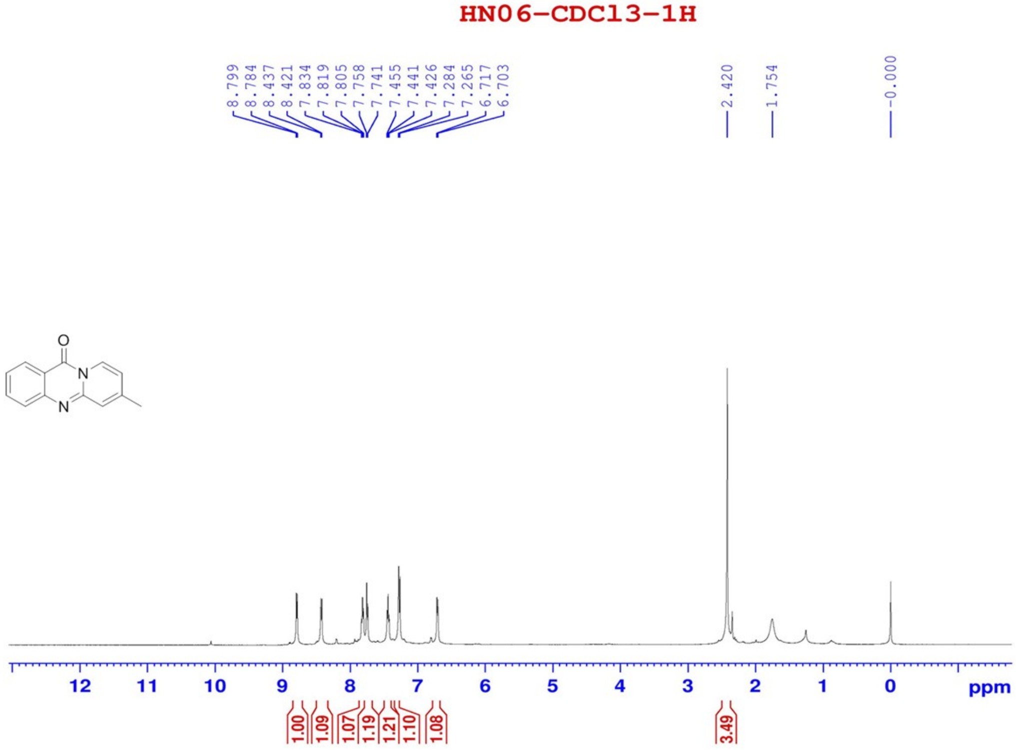


Fig.S5. $^{1}$H-NMR spectra of 7-methyl-11*H*-pyrido[2,1-*b*]quinazolin-11-one.

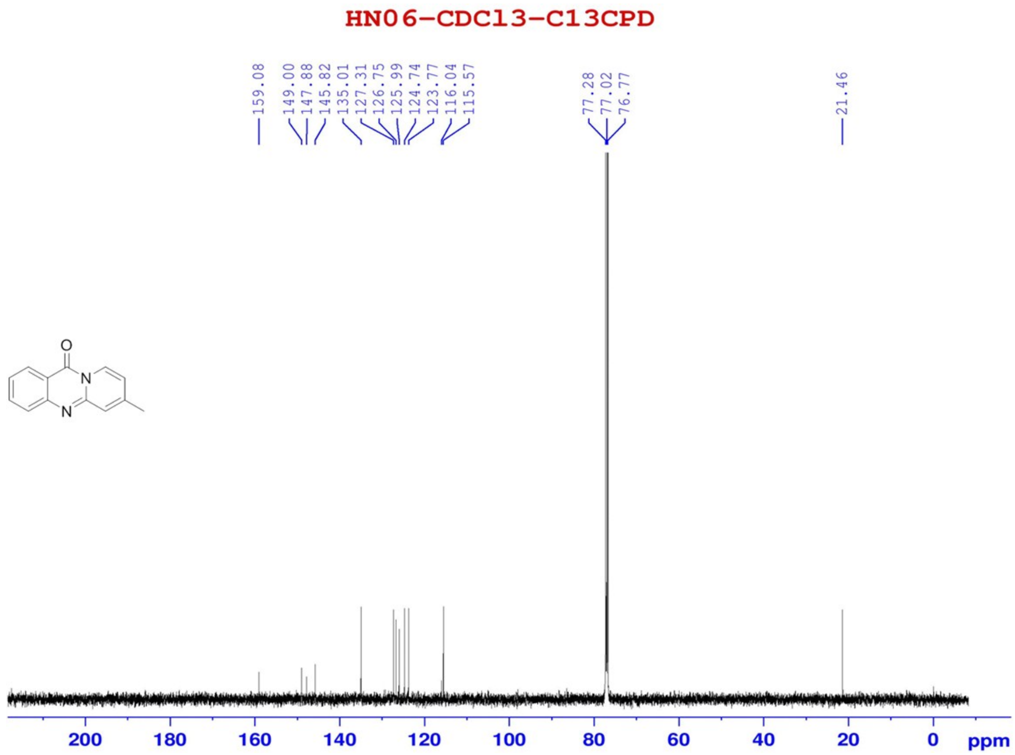


Fig.S6. $^{13}$C-NMR spectra of 7-methyl-11*H*-pyrido[2,1-*b*]quinazolin-11-one.

**Characterization data for 7-methyl-11*H*-pyrido[2,1-*b*]quinazolin-11-one**

Prepared as shown in the general experimental procedure and purified on silica gel (230-400 mesh or 37-63 μm, ethyl acetate/hexane = 1:1 (v./v.), TLC silica gel 60 $F_{254}$, $R_f$ = 0.45): Yellow solid, 72% yield (15 mg). $^{1}$H-NMR (500 MHz, $CDCl_3$) δ(ppm) 2.42 (s, 3H), 6.70 (d, J = 7.5 Hz, 1H), 7.27 (d, J = 9.5 Hz, 1H), 7.44 (t, J = 7.5 Hz, 1H), 7.75 (d, J = 8.5 Hz, 1H), 7.81 (t, J = 7.5 Hz, 1H), 8.43 (d, J = 9.5 Hz, 1H), 8.79 (d, J = 7.5 Hz, 1H) .$^{13}$C NMR ($CDCl_3$, 125 MHz) δ(ppm) 21.4, 115.5, 116.0, 123.7, 124.7, 125.9, 126.7, 127.3, 135.0, 145.8, 147.8, 149.0, 159.0.

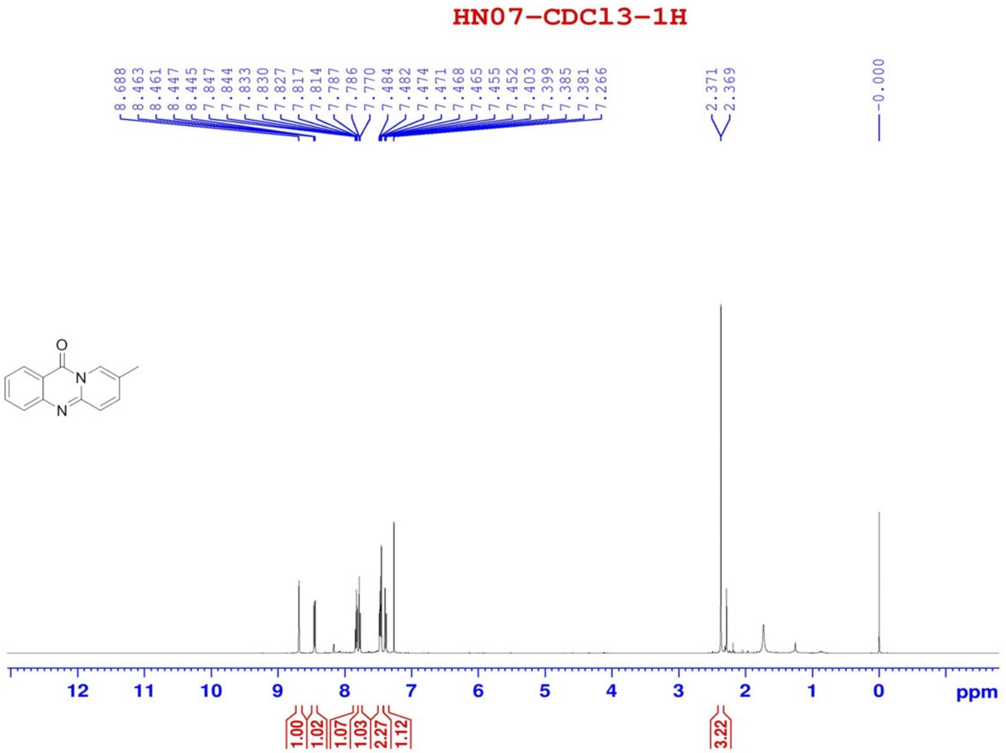


Fig.S7. $^{1}$H-NMR spectra of 8-methyl-11*H*-pyrido[2,1-*b*]quinazolin-11-one.

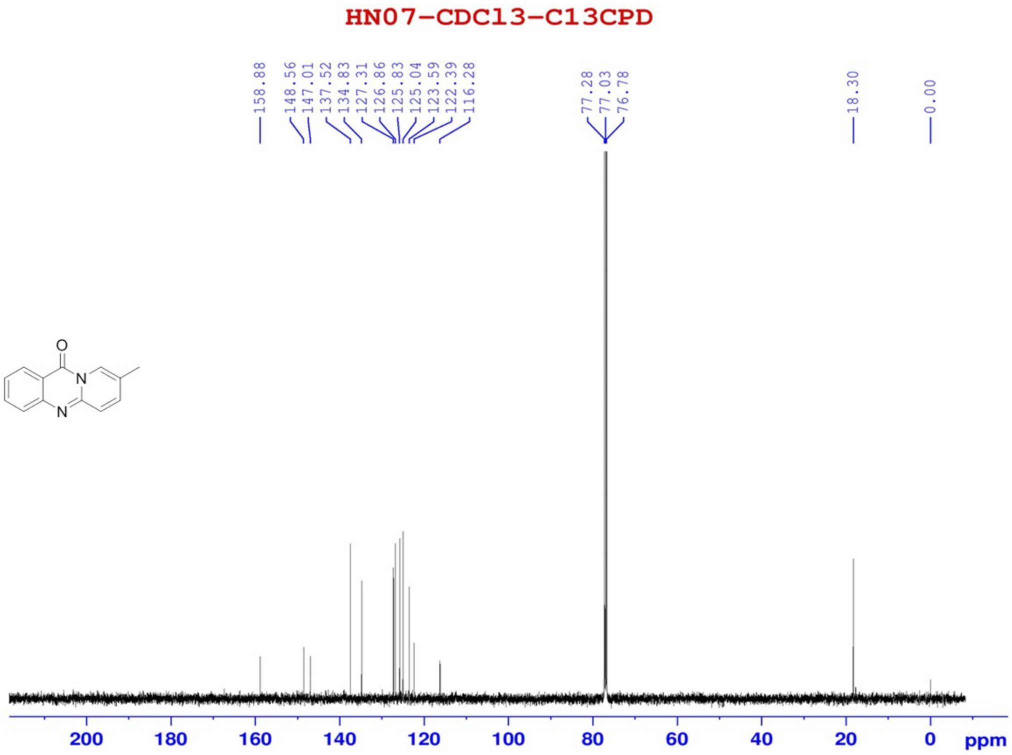

Fig.S8. $^{13}$C-NMR spectra of 8-methyl-11*H*-pyrido[2,1-*b*]quinazolin-11-one.

**Characterization data for 8-methyl-11*H*-pyrido[2,1-*b*]quinazolin-11-one**

Prepared as shown in the general experimental procedure and purified on silica gel (230-400 mesh or 37-63 μm, ethyl acetate/hexane = 1:1 (v./v.), TLC silica gel 60 $F_{254}$, $R_f$ = 0.45): Yellow solid, 82% yield (17 mg). $^{1}$H-NMR (500 MHz, $CDCl_3$) δ(ppm) 2.37 (d, J = 1 Hz, 3H), 7.39 (dd, J = 9 Hz, 2 Hz, 1H), 7.45–7.48 (m, 2H), 7.78 (dd, J = 8.5 Hz, 0.5 Hz, 1H), 7.83 (td, J = 7.0 Hz, 1.5 Hz, 1H), 8.45 (dd, J = 8.0 Hz, 1.0 Hz, 1H), 8.68 (s, 1H). $^{13}$C NMR ($CDCl_3$, 125 MHz) δ(ppm) 18.3, 116.2, 122.3, 123.5, 125.0, 125.8, 126.8, 127.3, 134.8, 137.5, 147.0, 148.5, 158.8.

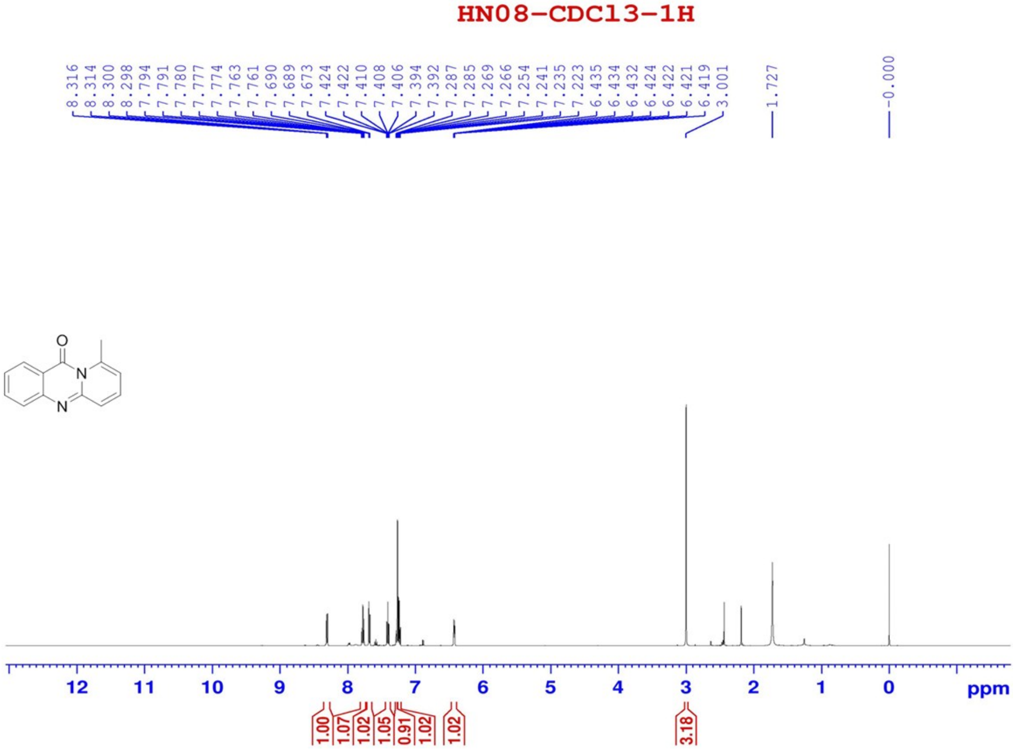


Fig.S9. $^{1}$H-NMR spectra of 9-methyl-11*H*-pyrido[2,1-*b*]quinazolin-11-one.

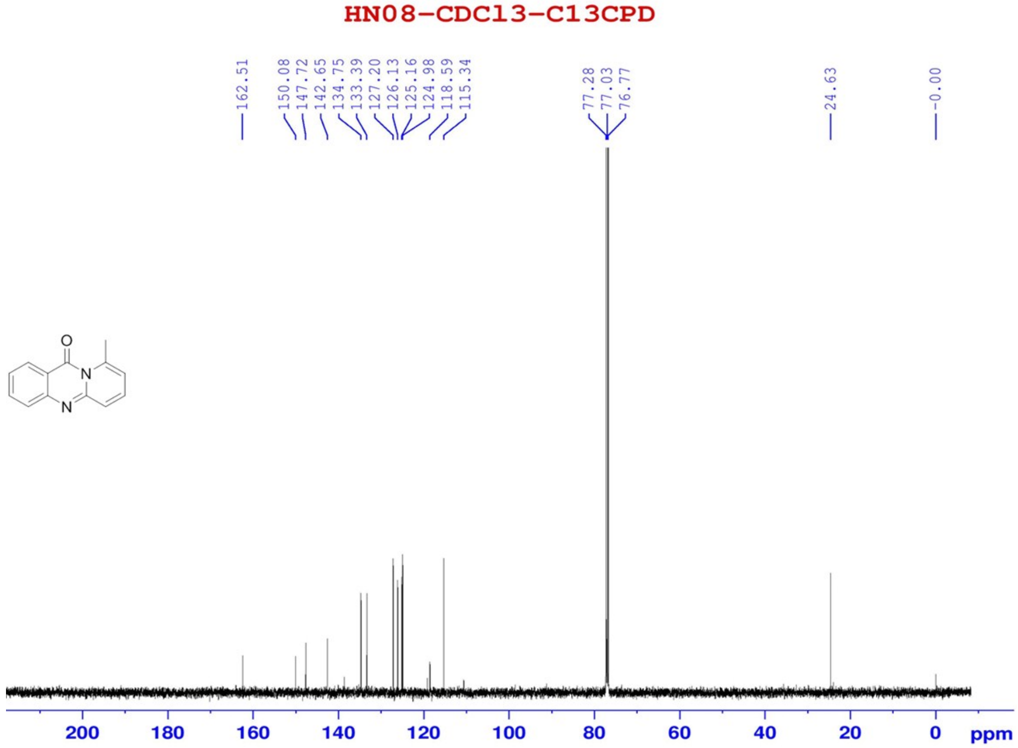


Fig.S10. $^{13}$C-NMR spectra of 9-methyl-11*H*-pyrido[2,1-*b*]quinazolin-11-one.

**Characterization data for 9-methyl-11*H*-pyrido[2,1-*b*]quinazolin-11-one**

Prepared as shown in the general experimental procedure and purified on silica gel (230-400 mesh or 37-63 μm, ethyl acetate/hexane = 1:1 (v./v.), TLC silica gel 60 $F_{254}$, $R_f$ = 0.45): Yellow solid, 71% yield (15 mg). $^1$H-NMR (500 MHz, $CDCl_3$) δ(ppm) 3.00 (s, 3H), 6.43 (dt, J = 6.5 Hz, 1.0 Hz, 1H), 7.22–7.28 (m, 2H), 7.40 (td, J = 7.0 Hz, 1.0 Hz, 1H), 7.68 (dd, J = 8.5 Hz, 0.5 Hz, 1H), 7.79 (td, J = 8.5 Hz, 1.5 Hz, 1H), 8.31 (dd, J = 8.5 Hz, 1.0 Hz, 1H). $^{13}$C NMR ($CDCl_3$, 125 MHz) δ(ppm) 24.6, 115.3, 118.5, 124.9, 125.1, 126.1, 127.2, 133.3, 134.7, 142.6, 147.7, 150.0, 162.5.

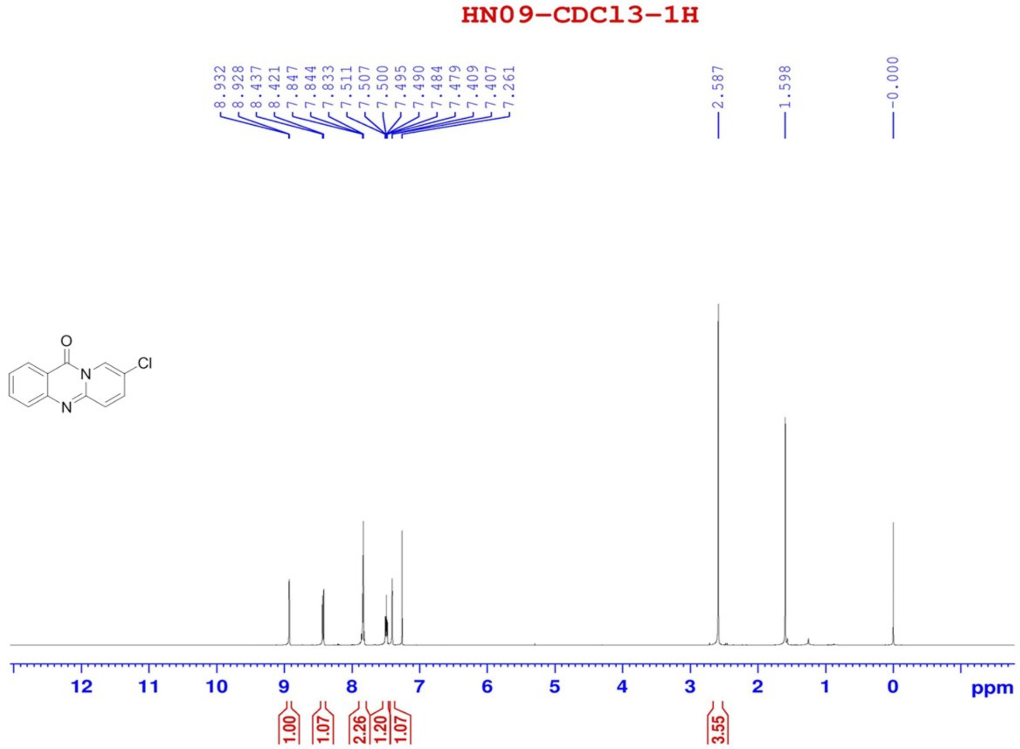

Fig.S11. $^{1}$H-NMR spectra of 8-chloro-11*H*-pyrido[2,1-*b*]quinazolin-11-one.

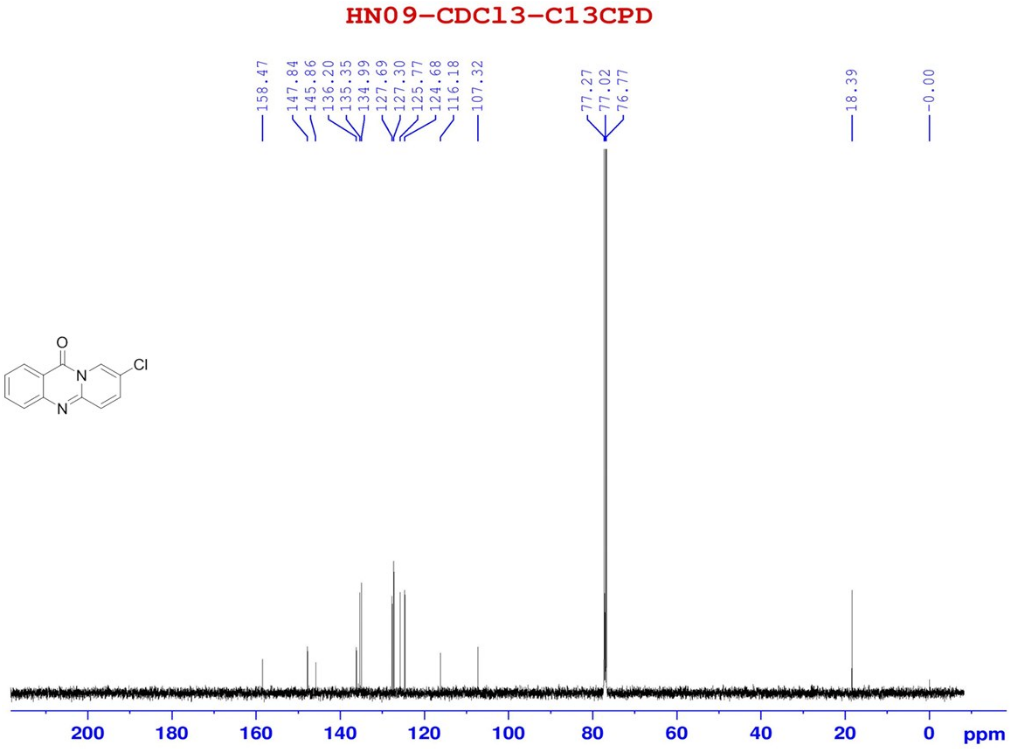


Fig.S12. $^{13}$C-NMR spectra of 8-chloro-11*H*-pyrido[2,1-*b*]quinazolin-11-one.

**Characterization data for 8-chloro-11*H*-pyrido[2,1-*b*]quinazolin-11-one**

Prepared as shown in the general experimental procedure and purified on silica gel (230-400 mesh or 37-63 μm, ethyl acetate/hexane = 1:1 (v./v.), TLC silica gel 60 $F_{254}$, $R_f$ = 0.4): Yellow solid, 55% yield (16 mg). $^{1}$H-NMR (500 MHz, $CDCl_3$) δ(ppm) 2.58 (s, 3H), 7.40 (s, 1H), 7.47–7.51 (m, 1H), 7.83–7.84 (m, 2H), 8.43 (d, J = 8.0 Hz, 1H), 8.93 (d, J = 2.0 Hz, 1H). $^{13}$C NMR ($CDCl_3$, 125 MHz) δ(ppm) 18.3, 107.3, 116.1, 124.6, 125.7, 127.3, 127.6, 134.9, 135.3, 136.2, 145.8, 147.8, 158.4.

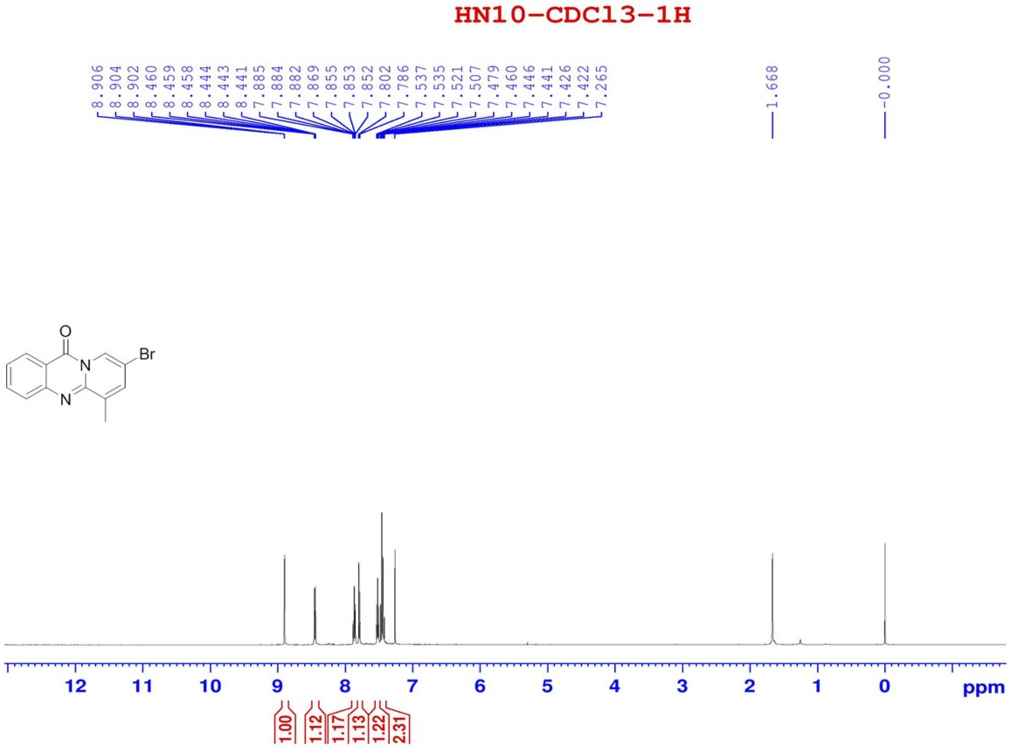


Fig.S13. $^{1}$H-NMR spectra of 8-bromo-6-methyl-11*H*-pyrido[2,1-*b*]quinazolin-11-one.

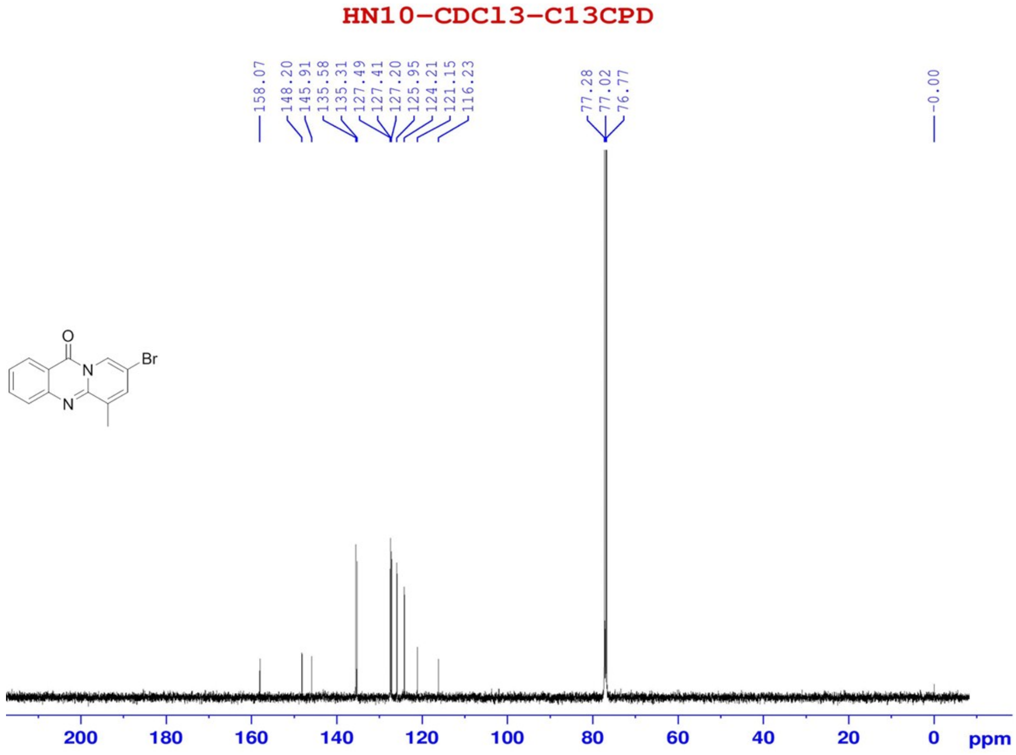


Fig.S14. $^{13}$C-NMR spectra of 8-bromo-6-methyl-11*H*-pyrido[2,1-*b*]quinazolin-11-one.

**Characterization data for 8-bromo-6-methyl-11*H*-pyrido[2,1-*b*]quinazolin-11-one**

Prepared as shown in the general experimental procedure and purified on silica gel (230-400 mesh or 37-63 μm, ethyl acetate/hexane = 1:1 (v./v.), TLC silica gel 60 $F_{254}$, $R_f$ = 0.4): Yellow solid, 74% yield (17 mg). $^1$H-NMR (500 MHz, $CDCl_3$) δ(ppm) 7.42–7.47 (m, 2H), 7.86 (t, J = 7.0 Hz, 1H), 7.79 (d, J = 8.0 Hz, 1H), 7.86 (td, J = 7.5 Hz, 0.5 Hz, 1H), 8.45 (dt, J = 8.0 Hz, 0.5 Hz, 1H), 8.90–8.91 (m, 1H). $^{13}$C NMR ($CDCl_3$, 125 MHz) δ(ppm) 116.2, 121.1, 124.2, 125.9, 127.2, 127.4, 127.5, 135.3, 135.5, 145.9, 148.2, 158.0.

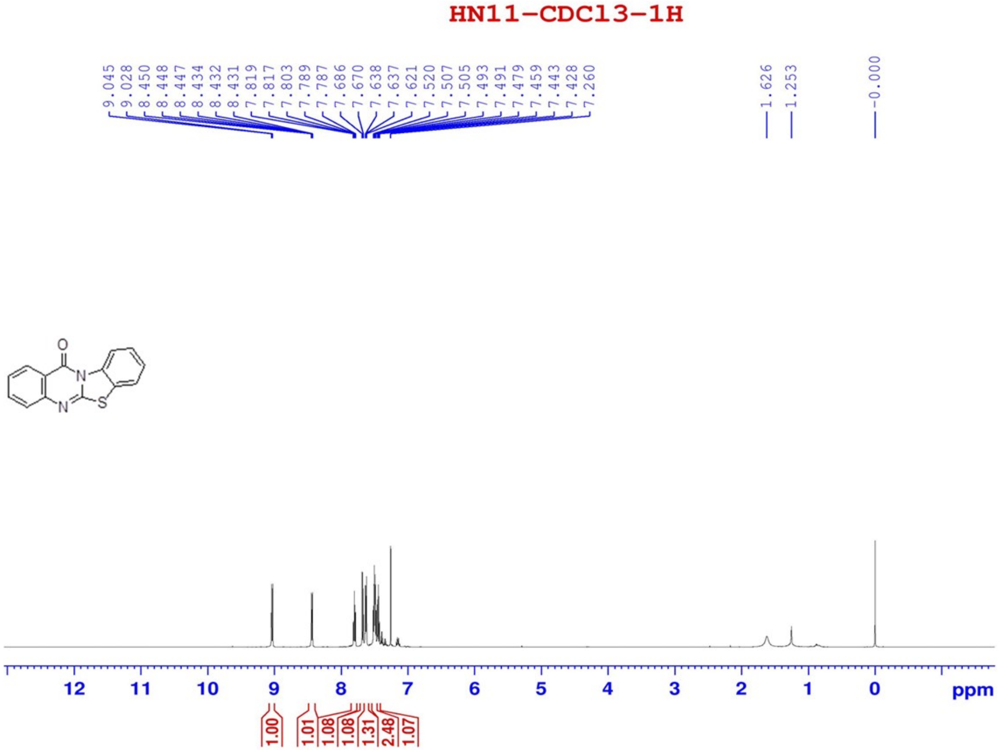


Fig.S15. $^{1}$H-NMR spectra of 12*H*-benzo[4,5]thiazolo[2,3-*b*]quinazolin-12-one.

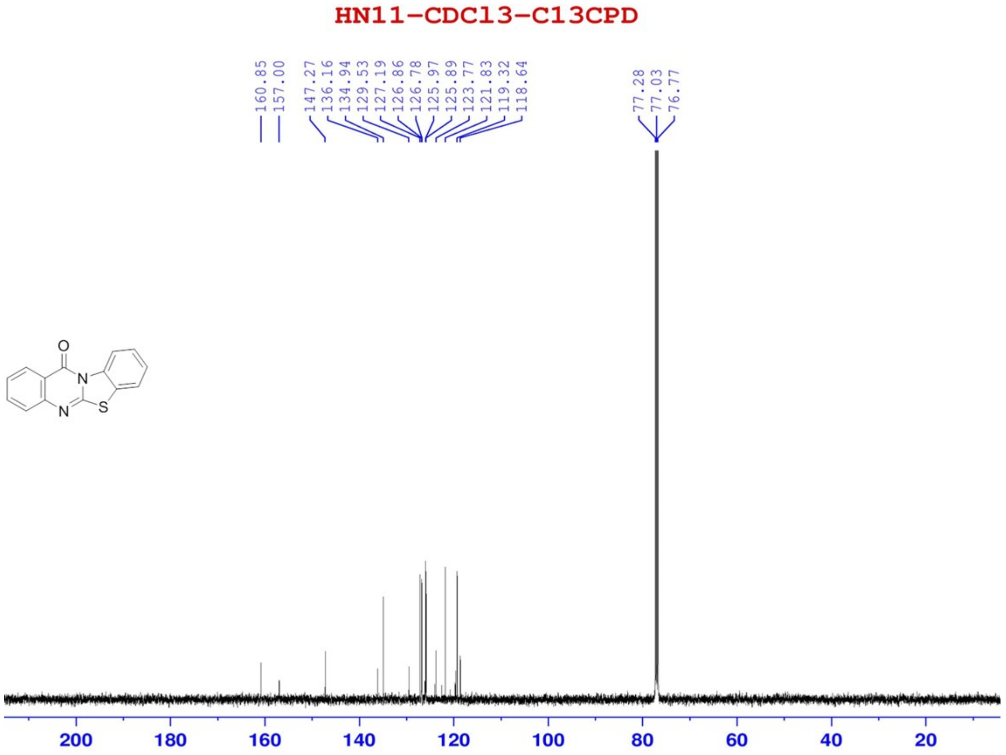


Fig.S16. $^{13}$C-NMR spectra of 12*H*-benzo[4,5]thiazolo[2,3-*b*]quinazolin-12-one.

**Characterization data for 12*H*-benzo[4,5]thiazolo[2,3-*b*]quinazolin-12-one**

Prepared as shown in the general experimental procedure and purified on silica gel (230-400 mesh or 37-63 μm, ethyl acetate/hexane = 1:2 (v./v.), TLC silica gel 60 $F_{254}$, $R_f$ = 0.35): Yellow solid, 57% yield (15 mg). $^1$H-NMR (500 MHz, $CDCl_3$) δ(ppm) 7.44 (t, J = 8.0 Hz, 1H), 7.47–7.52 (m, 2H), 7.63 (d, J = 8.5 Hz, 1H), 7.68 (d, J = 8.0 Hz, 1H), 7.80 (t, J = 8.0 Hz, 1H), 8.44 (d, J = 8.0 Hz, 1H), 9.03 (d, J = 8.5 Hz, 1H). $^{13}$C NMR ($CDCl_3$, 125 MHz) δ(ppm) 118.6, 119.3, 121.8, 123.7, 125.8, 125.9, 126.7, 126.8, 127.1, 129.5, 134.9, 136.1, 147.2, 160.8.

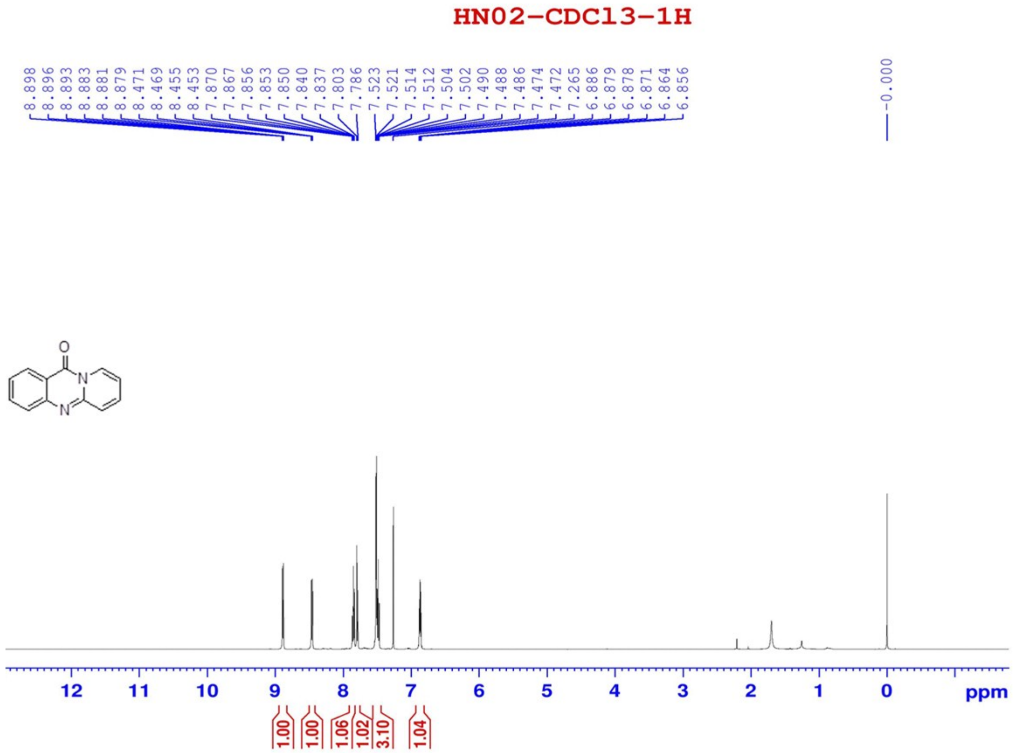


Fig.S17. $^{1}$H-NMR spectra of 11*H*-pyrido[2,1-*b*]quinazolin-11-one.

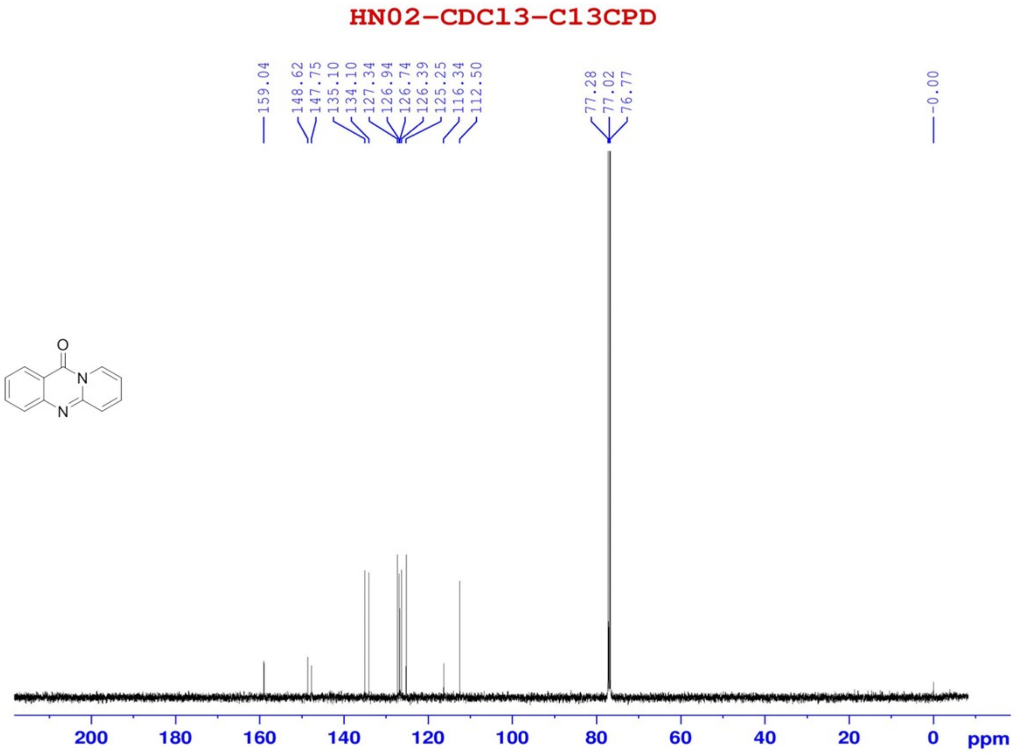


Fig.S18. $^{13}$C-NMR spectra of 11*H*-pyrido[2,1-*b*]quinazolin-11-one.

**Characterization data for 11*H*-pyrido[2,1-*b*]quinazolin-11-one**

Prepared as shown in the general experimental procedure and purified on silica gel (230-400 mesh or 37-63 μm, ethyl acetate/hexane = 1:1 (v./v.), TLC silica gel 60 $F_{254}$, $R_f$ = 0.4): Yellow solid, 89% yield (17 mg).$^1$H-NMR (500 MHz, $CDCl_3$) δ(ppm) 6.85–6.88 (m, 1H), 7.47–7.52 (m, 3H), 7.79 (d, J = 8.0 Hz, 1H), 7.83–7.86 (m, 1H), 8.46 (dd, J = 8.5 Hz, 1.0 Hz, 1H), 8.88 (dt, *J* = 8.5 Hz, 1.0 Hz, 1H).$^{13}$C NMR ($CDCl_3$, 125 MHz) δ(ppm) 112.5, 116.3, 125.2, 126.3, 126.7, 126.9, 127.3, 134.1, 135.1, 147.7, 148.6, 159.0

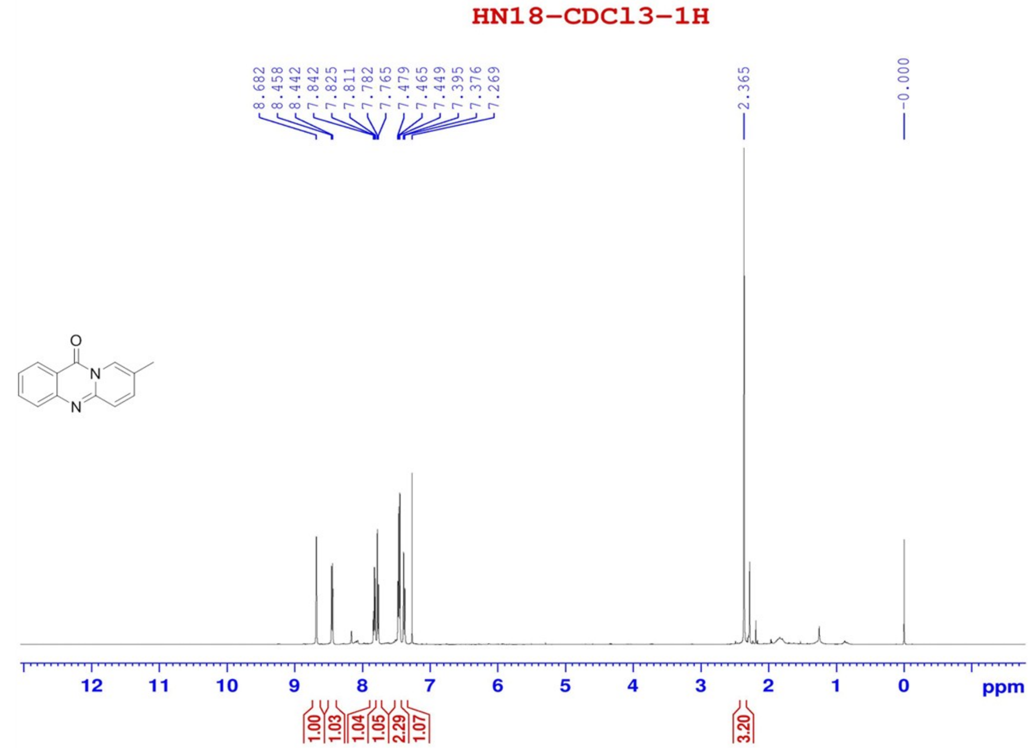


Fig.S19. $^{1}$H-NMR spectra of 8-methyl-11*H*-pyrido[2,1-*b*]quinazolin-11-one.

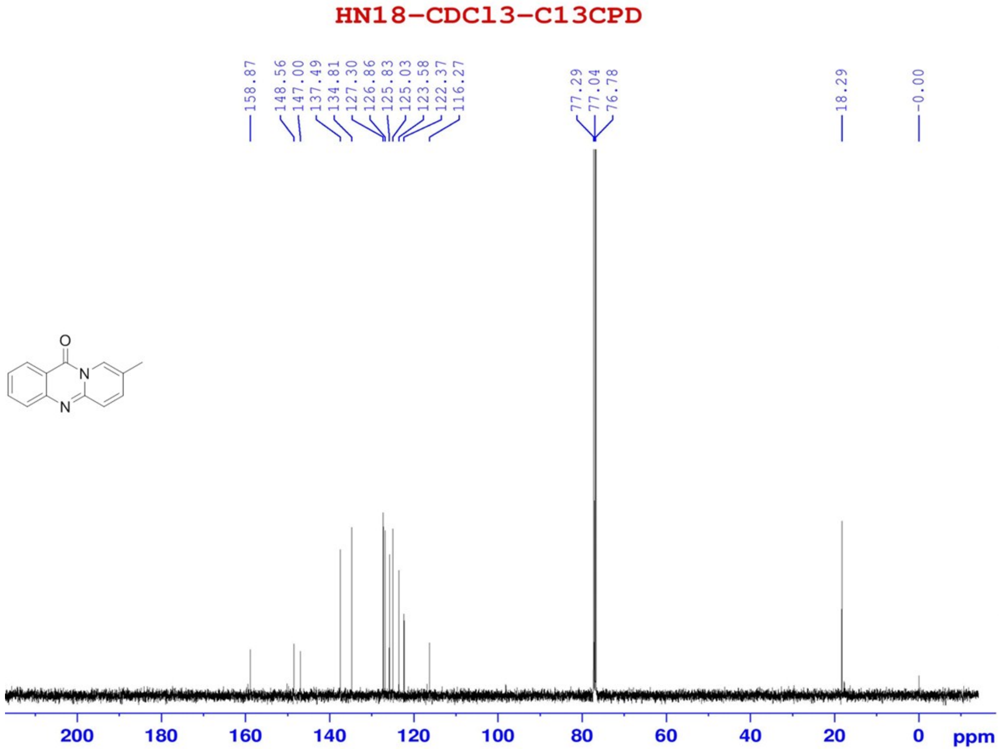


Fig.S20. $^{13}$C-NMR spectra of 8-methyl-11*H*-pyrido[2,1-*b*]quinazolin-11-one.

**Characterization data for 8-methyl-11*H*-pyrido[2,1-*b*]quinazolin-11-one**

Prepared as shown in the general experimental procedure and purified on silica gel (230-400 mesh or 37-63 μm, ethyl acetate/hexane = 1:1 (v./v.), TLC silica gel 60 $F_{254}$, $R_f$ = 0.45): Yellow solid, 87% yield (18 mg). $^1$H-NMR (500 MHz, $CDCl_3$) δ(ppm) 2.36 (s,3H), 7.38 (d, J = 9.5 Hz, 1H), 7.45 (d, J = 8.0 Hz, 2H), 7.77 (d, J = 8.5 Hz, 1H), 7.81 – 7.84 (m, 1H), 8.45 (d, J = 8.0 Hz, 1H), 8.68 (s, 1H). $^{13}$C NMR ($CDCl_3$, 125 MHz) δ(ppm) 18.2, 116.2, 122.3, 123.5, 125.0, 125.8, 126.8, 127.3, 134.8, 137.4, 147.0, 148.5, 158.8

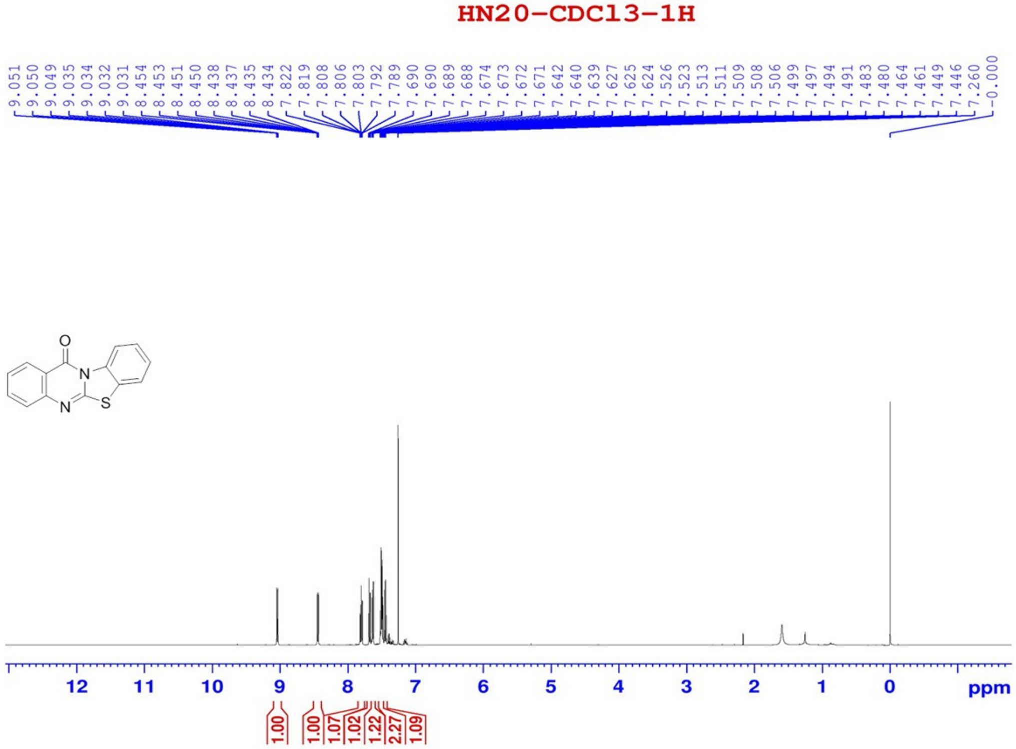


Fig.S21. $^{1}$H-NMR spectra of 12*H*-benzo[4,5]thiazolo[2,3-*b*]quinazolin-12-one.

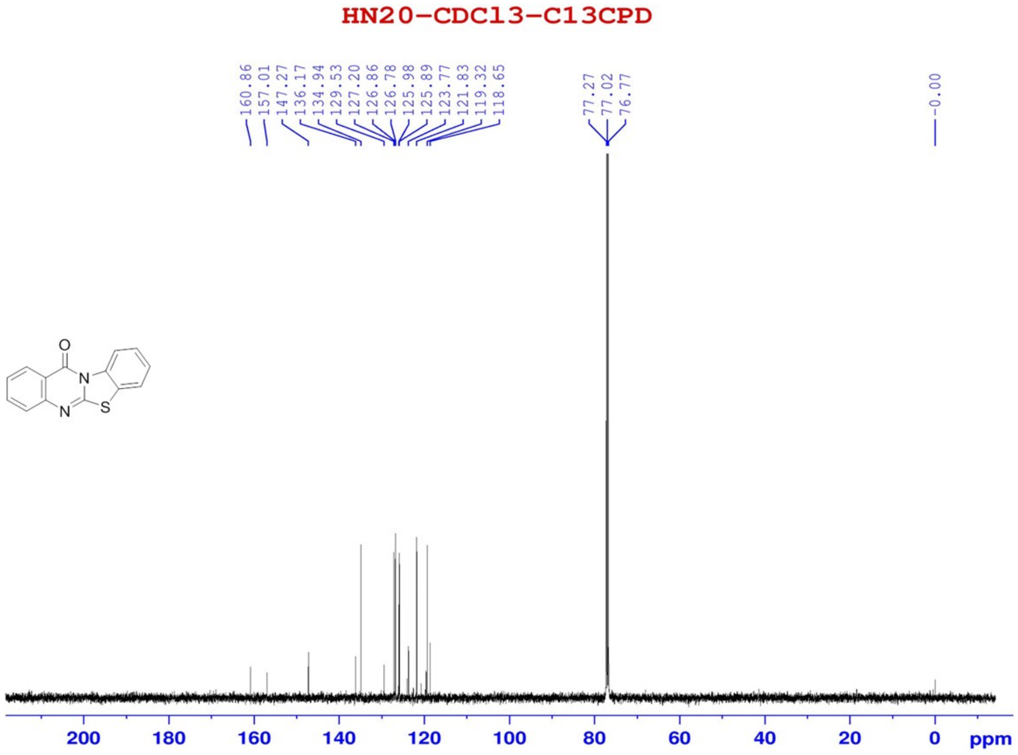


Fig.S22. $^{13}$C-NMR spectra of 12*H*-benzo[4,5]thiazolo[2,3-*b*]quinazolin-12-one.

**Characterization data for 12*H*-benzo[4,5]thiazolo[2,3-*b*]quinazolin-12-one**

Prepared as shown in the general experimental procedure and purified on silica gel (230-400 mesh or 37-63 μm, ethyl acetate/hexane = 1:1 (v./v.), TLC silica gel 60 $F_{254}$, $R_f$ = 0.35): Yellow solid, 63% yield (16.5 mg). $^{1}$H-NMR (500 MHz, $CDCl_3$) δ(ppm) 7.44 (td, J = 7.5 Hz, 1.5 Hz, 1H), 7.48 – 7.52 (m, 2H), 7.62 – 7.64 (m, 1H), 7.67 – 7.69 (m, 1H), 7.78 – 7.82 (m, 1H), 9.03 – 9.05 (m, 1H). $^{13}$C NMR ($CDCl_3$, 125 MHz) δ(ppm) 118.6, 119.3, 121.8, 123.7, 125.8, 125.9, 126.7, 126.8, 127.2, 134.9, 136.1, 147.2, 157.0, 160.8

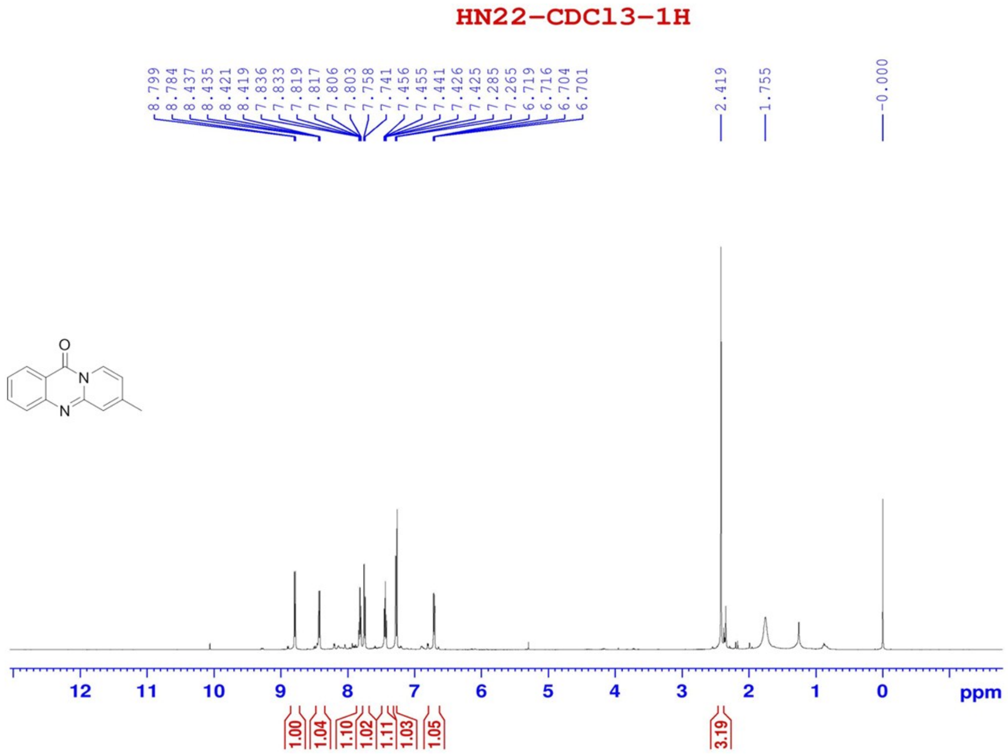


Fig.S23. $^{1}$H-NMR spectra of 7-methyl-11*H*-pyrido[2,1-*b*]quinazolin-11-one.

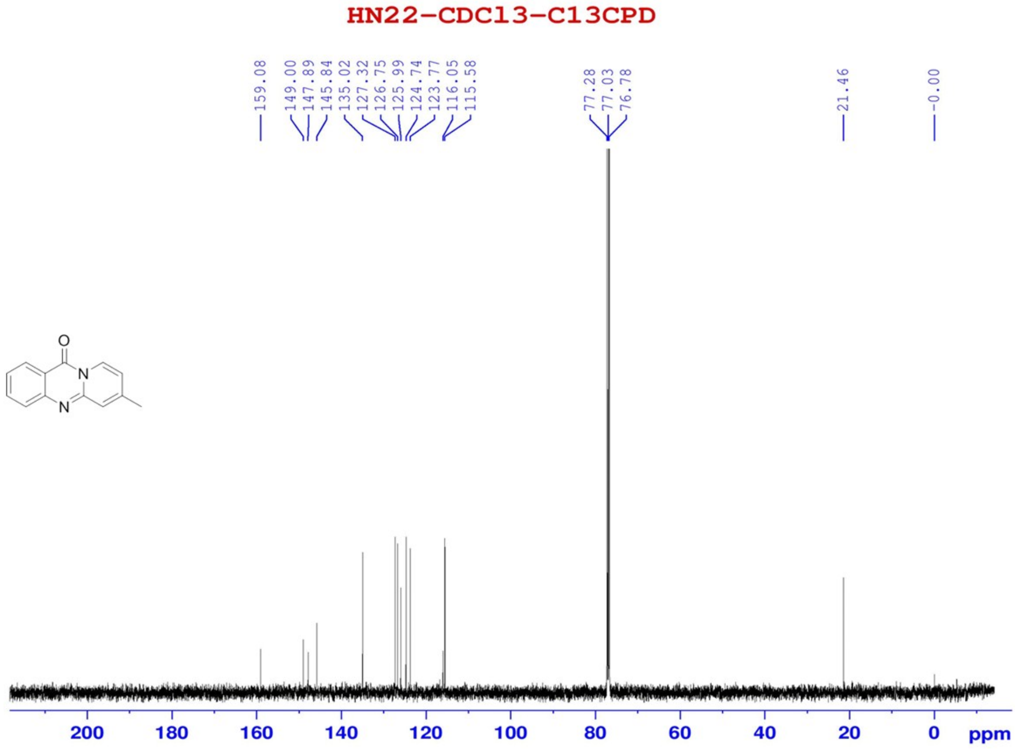


Fig.S24. $^{13}$C-NMR spectra of 7-methyl-11*H*-pyrido[2,1-*b*]quinazolin-11-one.

**Characterization data for 7-methyl-11*H*-pyrido[2,1-*b*]quinazolin-11-one (HN22 – Entry 12)**

Prepared as shown in the general experimental procedure and purified on silica gel (230-400 mesh or 37-63 μm, ethyl acetate/hexane = 1:1 (v./v.), TLC silica gel 60 $F_{254}$, $R_f$ = 0.45): Yellow solid, 78% yield (16.3 mg).$^1$H-NMR (500 MHz, $CDCl_3$) δ(ppm) 2.41 (s,3H), 6.70 (d, J = 1.5 Hz, 1H), 7.26 – 7.28 (m, 1H), 7.44 (t, J = 7.5 Hz, 1H), 7.75 (d, J = 9.0 Hz, 1H), 7.81 (td, J = 9.0 Hz, 1.5 Hz, 1H), 8.43 (dd, J = 7.5 Hz, 1Hz, 1H), 8.79 (d, J = 7.5 Hz, 1H). $^{13}$C NMR ($CDCl_3$, 125 MHz) δ(ppm) 21.4, 115.5, 116.0, 123.7, 124.7, 125.9, 126.7, 127.3, 135.0, 145.8, 147.8, 149.0, 159.0

**Table S8: Effect of strong electron-withdrawing substituents on 2-aminopyridine[a]**

| Entry | Reactant 1 | Reactant 2 | Product | Isolated Yield (%) |
|---|---|---|---|---|
| 1 | 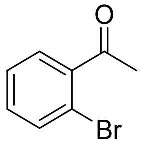 | 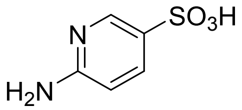 | 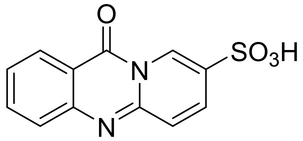 | Trace |
| 2 | 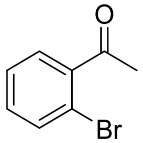 | 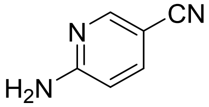 | 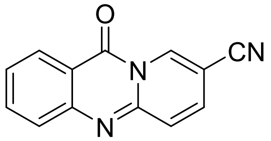 | Trace |
| 3 | 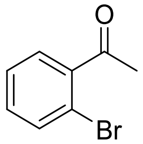 | 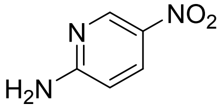 | 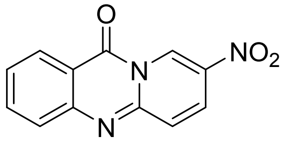 | Trace |
| 4 | 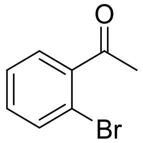 | 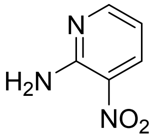 | 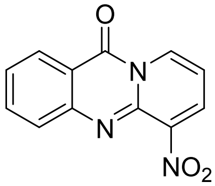 | Trace |

[a] [a]Reaction conditions: 2'-bromoacetophenone (0.1 mmol); 2-aminopyridines (0.25 mmol); NaOAc (0.2 mmol); DMSO (0.5 mL); $Cu(OAc)_2.H_2O$ catalyst (20 mol%); oxygen atmosphere; 120 $^oC$; 4 h.
